# Antimicrobial Activity of the Secondary Metabolites Isolated from a South African Red Seaweed, *Laurencia corymbosa*

**DOI:** 10.3390/molecules28052063

**Published:** 2023-02-22

**Authors:** Jameel Fakee, John J. Bolton, Marilize Le Roes-Hill, Kim A. Durrell, Edith Antunes, Denzil R. Beukes

**Affiliations:** 1 Faculty of Pharmacy, Rhodes University, Makhanda 6140, South Africa; 2 Department of Biological Sciences, University of Cape Town, Rondebosch 7701, South Africa; 3 Applied Microbial and Health Biotechnology Institute, Cape Peninsula University of Technology, Bellville 7535, South Africa; 4 Department of Chemistry, University of the Western Cape, Bellville 7535, South Africa; 5 School of Pharmacy, University of the Western Cape, Bellville 7535, South Africa

**Keywords:** *Laurencia corymbosa*, cuparanes, chamigranes, antimicrobial activity

## Abstract

South Africa’s highly diverse marine biota includes several endemic marine red algae of the *Laurencia* genus. Cryptic species and morphological variability make the taxonomy of *Laurencia* plant challenging, and a record of the secondary metabolites isolated from South African *Laurencia* spp. can be used to assess their chemotaxonomic significance. In addition, the rapid development of resistance against antibiotics, coupled with the inherent ability of seaweeds to resist pathogenic infection, supported this first phycochemical investigation of *Laurencia corymbosa* J. Agardh. A new tricyclic keto-cuparane (**7**) and two new cuparanes (**4, 5**) were obtained alongside known acetogenins, halo-chamigranes, and additional cuparanes. These compounds were screened against *Acinetobacter baumannii*, *Enterococcus faecalis*, *Escherichia coli*, *Staphylococcus aureus*, and *Candida albicans*, with **4** exhibiting excellent activity against the Gram-negative *A. baumanii* (minimum inhibitory concentration (MIC) 1 μg/mL) strain.

## 1. Introduction

The increasing number of marine-derived therapeutic agents justifies the continued exploration of the oceans as a source of new medicines [1]. To date, more than 20 drugs from the marine environment have been approved for use. In 1969, the FDA approved cytarabine (Cytosar-U^®^), isolated from the Caribbean sponge *Cryptotheca crypta*, for use as an anticancer drug. Since then, the analgesic cone-snail-derived peptide ziconotide (Prialt^®^) and the anticancer sponge-derived macrolide eribulin mesylate (Halaven^®^) as well as other natural products with anticancer (Fludarabine, Nelarabine, Keyhole Limpet Hemocyanin K, Protamine Sulfate, Lurbinectedin, Brentuximab vedotin etc.), antiviral (Vidarabine), and antihypertriglyceridemia (Eicosapentaenoic acid ethyl ester, Omega-3-carboxylic acid) activities have been approved [2,3,4,5,6,7]. In addition, propylene glycol alginate sulfate sodium (PSS) and propylene glycol mannuronate sulfate (PGMS) isolated from macroalgae have been approved for use in cardiovascular and hyperlipidemia disease areas [8,9]. Currently, there are more than 50 drug candidates in different stages of clinical trials, with more than 85% undergoing trials as anticancer agents [2,3,4,5,6,7]. However, there are very few anti-infective agents that have been approved or are undergoing clinical trials. Economic and regulatory obstacles have effectively halted the development of new antibiotics, while funding cuts have resulted in a decrease in academic research. The development of antibiotics is not considered economically sound since antibiotics are often curative and used for relatively short periods, i.e., antibiotics are not as profitable as drugs that treat chronic conditions, such as diabetes and asthma. All these factors have led to the antibiotic resistance crisis [10].

Marine macroalgae (seaweeds) have generated over 3000 biologically active, new chemical structures in the last 40 years, most of which were isolated from the phylum Rhodophyta [11]. More than 1200 unique natural products, including halogenated sesquiterpenes, diterpenes, C15 acetogenins, and indoles, have been reported from the red algal genus *Laurencia* [12,13]. Unfortunately, factors such as high morphological variability amongst individual species make the classification and/or identification of members within this genus increasingly difficult [14].

Marine organisms, including seaweeds, are constantly exposed to potentially pathogenic microorganisms but are rarely infected. It is thus hypothesized that seaweeds produce small molecules that act as antimicrobial substances and prevent infection [15]. Seaweed natural products may thus provide lead compounds in the discovery of new antibiotics. Fortunately, natural products remain the leading source of new antibiotics against pathogenic microorganisms [16,17].

**Figure 1:**
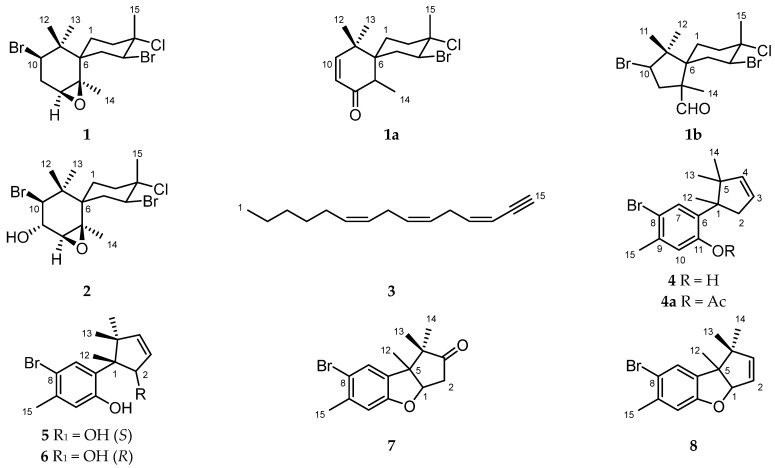
Compounds **1**–**8** isolated (and derivatives produced) from the South African *Laurencia corymbosa* sp.

The purpose of the current study is therefore two-fold: (1) to document, for the first time, the secondary metabolites of *L. corymbosa*, which may assist in the taxonomic classification of species of *Laurencia*, and (2) to assess their antimicrobial potential. In this first phycochemical analysis of South African *Laurencia corymbosa*, we report the isolation, structural characterization, and antimicrobial activity of the known chamigranes (**1** and **2**), the C15 acetogenin neolaurencenyne (**3**), and new cuparanes (**4, 5,** and **7**) (Figure 1) [18].

## 2. Results and Discussion

**Figure 2:**
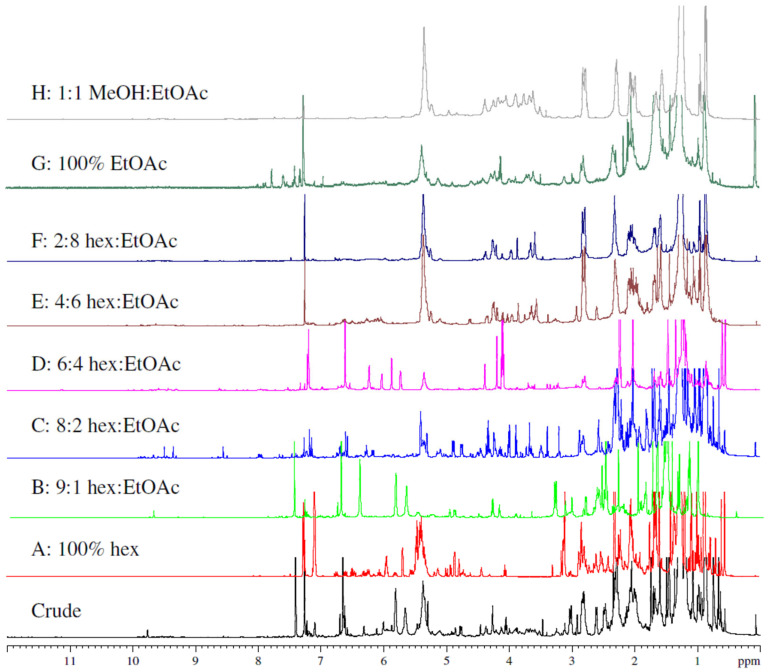
^1^H NMR spectra (CDCl_3_, 600 MHz) of the crude organic extract of *L. corymbosa* and the column fractions A–H. Abbreviations: Hex: hexane; EtOAc: ethyl acetate; MeOH: methanol.

Specimens of *L. corymbosa* were extracted with MeOH and MeOH-CH_2_Cl_2_, and the combined extracts were concentrated at 40 °C. Column chromatography on silica gel using hexane-EtOAc-MeOH solvent combinations of increasing polarity resulted in eight fractions (frs A-H, Scheme S1). The effectiveness of this extraction and separation protocol in separating different polarity classes and concentrating minor natural products is easily seen when comparing the ^1^H NMR spectra of the various fractions (Figure 2). Further fractionation and purification by normal phase HPLC resulted in the isolation of compounds **1**–**8**.

### 2.1. Chamigranes

Compound **1**, crystalline solid, was purified by normal phase HPLC of column fr B (hexane-EtOAc, 9:1).

The ^1^H NMR spectrum (Figure S2) of the crystalline material together with a multiplicity-edited HSQC spectrum (Figure S5) were key in identifying three sets of diastereotopic methylene resonances at δ_H_ 1.67 and 1.98, δ_H_ 2.38 and 2.47, δ_H_ 2.10 and 2.84, and one enantiotopic methylene resonance at δ_H_ 2.61 (dd, *J* = 7.7, 3.0 Hz). A total of three methine moieties observed at δ_H_ 4.77 (dd, *J* = 13.3, 4.7 Hz), δ_H_ 2.92 (t, *J* = 3.0 Hz) and δ_H_ 4.06 (t, *J* = 7.7 Hz), together with four methyl singlets between δ_H_ 1.0 and 2.0, were suggestive of a sesquiterpene skeleton. These data, together with the ^13^C and HMBC data obtained, supported a chamigrane sesquiterpene structure with a spiro [5.5] undecane arrangement for metabolites typically produced by *Laurencia* spp. [14].

**Figure 3:**
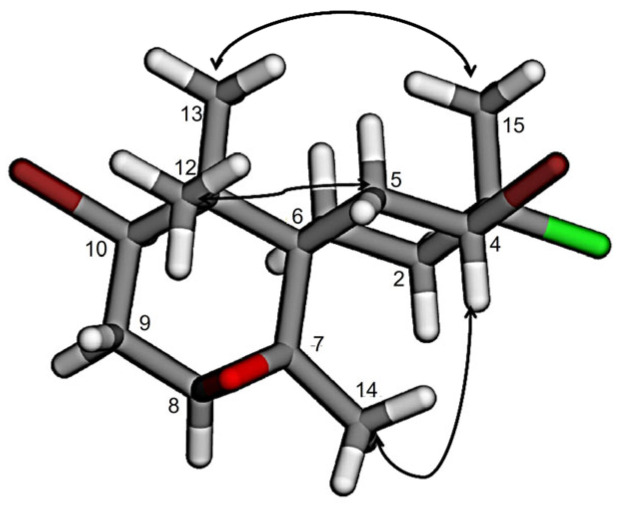
Key NOESY correlations for compound **1**.

**Figure 4:**
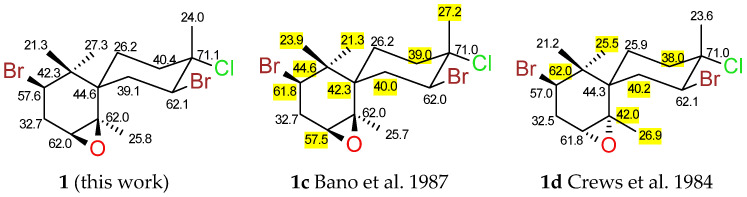
^13^C NMR shift comparisons for compound **1**. All δ_C_ chemical shifts reported in CDCl_3_ [22,23]. Chemical shifts in dispute are highlighted in yellow.

Compound **1** was identified as a previously isolated metabolite, 3-chloro-4,10-dibromo-7,8-epoxychamigrane, isolated from a *Laurencia* sp. in the Gulf of California [19], with the crystal structure reported by Cowe et al. (1989) [20]. Analysis of the NOESY spectrum of compound **1** allowed for the confident assignment of the stereo-centers as shown in Figure 3 and Figure S8. This allowed for a distinction to be made between compound **1** and the corresponding epoxy-epimer about C7/C8, compound **1d** (Figure 4), which was isolated by Ojika et al. (1982) [21] from *Laurencia okamurai*. The relative configuration displayed in compound **1** is further justified as ^1^H NMR data were consistent with those provided by Howard and Fenical (1975) [19].

Although compound **1** had previously been isolated from a *Laurencia* sp. in the Gulf of California [19], there is some conflict in the reported carbon chemical shifts [22] requiring an informed re-assignment following comprehensive analysis of the ^13^C NMR data of compound **1**. Unambiguous COSY (Figure S4) and HMBC (Figures S6 and S7) correlations obtained for compound **1** in this study conflicted with those reported by Bano et al. (1987) [22], as shown in compound **1** (Figure 4), especially regarding positions C6, C10, and C11. Although ^13^C NMR data are included by the authors (Ojika et al., 1982) [21] for the epoxy epimer **1d**, several discrepancies are observed (Figure 4). Crews et al. (1984) [23], however, report the ^13^C NMR data for a semi-synthetic analogue prepared by Howard and Fenical (1975) [19].

The C12 and C13 methyl protons show clear ^3^*J* HMBC correlations (Figures S6 and S7) to the bromo-methine at C10 where the chemical shift is δ_C_ 57.6 and not δ_C_ 61.8, as suggested by Bano et al. (1987), while the spiro-center at C6 in compound **1** possesses a quaternary carbon with a chemical shift of δ_C_ 44.6 and not δ_C_ 42.3 [22]. This is supported by unambiguous ^3^*J* HMBC correlations from methyl moieties at C13 and C14. In contrast to **1c**, the ^13^C NMR chemical shift of the gem-dimethyl quaternary carbon at C11 should be δ_C_ 42.3 and not δ_C_ 44.6, as both methyl groups C12 and C13 display strong ^2^*J* HMBC correlations to C11 (δ_C_ 42.3). Bano et al. (1987) [22] and Crews et al. (1984) [23] both report two carbon resonances at δ_C_ 62.0 (Figure 4).

**Scheme 1:**
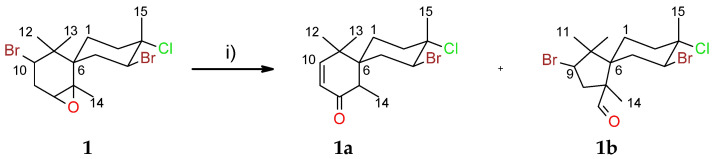
Epoxy cleavage of compound **1**. Conditions: (i) BF_3_ Et_2_O in anhydrous Et_2_O. Stirred for 18 h from −78 °C to RT (room temperature). NMR data are available in the supplementary information.

Comprehensive 2D analysis as well as a solvent change from CDCl_3_ to acetone failed to resolve the overlapped signals of C7 and C8. Cleavage of the epoxide with BF_3_·Et_2_O provided this confirmation through the formation of the dehydrohalogenated ketone **1a** and the ring-contracted cyclopentenyl aldehyde **1b** (Scheme 1). The formation of the α,β unsaturated ketone in **1a** was revealed by the ^1^H NMR spectrum (Figure S9, Table S1) with coupled (*J* = 10.2 Hz) olefinic doublets at δ_H_ 5.82 and δ_H_ 6.43, together with a carbonyl signal at δ_C_ 201.5 and a carbonyl stretch at 1681 cm^−1^ in the ^13^C NMR (Table S1) and IR data, respectively.

Compound **1b** showed a distinctive aldehyde singlet in its ^1^H NMR spectrum (Figure S10, Table S2) at δ_H_ 9.79, while the condensed five-membered ring was confirmed by examining ^2,3^*J* HMBC correlations within the pentacyclic ring from the methyl moieties positioned at C7 and C10. Additionally, the aldehyde functionality at C13 was clearly visible in the ^13^C NMR spectrum (Figure S11) of compound **1b** at δ_C_ 203.5. These data thus confirmed the integrity of the C7 assignment at δ_C_ 62.0.

The ^1^H NMR spectrum of compound **2** (Figure S12) indicated a similar chamigrane skeleton to that observed for compound **1**. Terpenoid shielded methyl moieties were apparent between δ_H_ 1.0 and δ_H_ 2.0, together with a cluster of methylene signals between δ_H_ 2.0 and 2.8. An additional methine signal in the range δ_H_ 3.7–5.0 suggested further substitution on the chamigrane skeleton of compound **2** vs. compound **1**. COSY and HMBC experiments revealed the presence of a methine moiety at C9 in **2** with the chemical shift at δ_C_ 70.7 supporting a hydroxyl moiety (Table S3, Figure S13). A coupling constant of (*J* = 9.1 Hz) suggested a trans-axial arrangement for the methine protons H9 and H10, while a coupling constant of *J* = 2.8 Hz for H9 allowed the assignment of the relative configuration at C7/C8. Elsworth and Thomson (1989) reported the isolation of compound **2** previously from *Laurencia glomerata* [24].

### 2.2. Linear Acetogenin

Compound **3** was isolated as a viscous nonpolar oil. The ^1^H NMR spectrum (Figure S14) contained a complex cluster of olefinic signals in the region of δ_H_ 5.0–6.0, suggesting unsaturated functionalities. A terminal methyl flanked by a methylene group was deduced from a broad triplet at δ_H_ 0.89 (t, *J* = 7.1 Hz), while a broad singlet at δ_H_ 3.11 identified a terminal alkyne proton. A further two double bonds (δ_C_ 125.8, 127.4, 129.9, and 130.6) and five methylene moieties (δ_C_ 28.7, 29.3, 31.5, 25.8, and 27.2) were deduced from a multiplicity edited HSQC spectrum (Table S4). Compound **3** was identified as neolaurencenyne, a non-terpenoid linear acetogenin previously isolated from *Laurencia okamurai* and successfully synthesized by Kigoshi et al. (1981) [25]. Compounds within this structural class are thought to be precursors to the more complex cyclic acetylenic compound. The orientation of the terminal alkyne about Δ^3,4^ was assigned as *cis,* as deduced by the carbon chemical shift at C2; with *cis* and *trans* systems resonating at ~δ_C_ 80 and ~δ_C_ 76, respectively [25]. The ^13^C NMR spectrum (Figure S15) exhibited 15 signals including resonances characteristic of a terminal conjugated alkenyne moiety (δ_C_ 81.8, 80.3, 108.3 and 143.7), which was further confirmed by an IR stretch at ~3300 cm^−1^.

A biosynthetically significant metabolite showing the precursor nature of **3** is the Δ^6,7^ hydroxy-chloro derivative of neolaurencenyne which was isolated by Norte et al. (1991) from a Spanish sample of *Laurencia pinnatifida* [26]. The latter compound hints at the enzyme-driven substitution of the olefin within **3**.

### 2.3. Cuparanes

Compound **4**, isolated as a colourless oil, showed a molecular ion cluster at *m/z* 294 and 296 in the HRGCEIMS spectrum (Figure S24), indicative of a single isotopic halo-substituent within the molecule. The molecular formula was deduced to be C_15_H_19_O^79^Br with a calculated double bond equivalent of six. The ^13^C NMR (Figure S18, Table 1), together with HSQC and HMBC data spectra, revealed 15 signals (Figures S20 and S21, respectively), as well as a broad hydroxy stretch in the IR spectrum which added integrity to the molecular formula. The ^1^H NMR spectrum (Figure S17) of **4** revealed four methyl resonances between δ_H_ 0.5 and 2.5, suggesting a sesquiterpenoid skeleton. The ^1^H NMR spectrum also showed aromatic singlets between δ_H_ 6.5 and 7.5, two deshielded, broad olefinic singlets between δ_H_ 5.5 and 6.5, and mutually coupled (*J* = 16.1 Hz) allylic methylene doublets at δ_H_ 2.5 and δ_H_ 3.0.

This new compound, compound **4**, was identified as an isomer of iso-allolaurinterol isolated by Dias et al. (2009) from *Laurencia filiformis* [27]. Iso-allolaurinterol shows a methyl shift from C5 to C4, whereas compound **4** exhibits the presence of a broad olefin singlet at C4 (δ_H_ 5.67) and a gem-dimethyl moiety at C5. Aromatic, hydroxyl substituents are known to be more deshielded than bromo-substituents [28]; thus, critical assessment of HMBC correlations from the methyl substituents at C1 and C9 to various carbon centers on the aromatic ring (Figures S21 and S22) allowed for the determination of the positions of the bromine and hydroxy moieties. Moreover, the proposed substituent locations in compound **4** are typical for metabolites isolated from *Laurencia* spp., as is shown by Dias et al. (2009), Yu et al. (2014), and Yamada et al. (1969) [27,29,30].

Acetylation of compound **4** to form **4a**, confirmed by acetyl carbon signals δ_C_ 169.6 and δ_C_ 21.8 (Figure S26, Table S5), resulted in clearer splitting of olefinic signals H3 and H4 as well as a more distinctive split for H2 (Figure S25). The HRGCEIMS spectrum (Figure S27) of the acetylated compound, **4a**, showed a molecular ion cluster [M+H]^+^ at *m/z* 337.0789 (calculated for 337.0803) and 339.0823, confirming the molecular formula C_17_H_21_^79^BrO_2_. Assignment of the positions of the C8 and C11 substituents in compound **4** were further substantiated as similar 2D correlations were observed in the acetylated derivative.

Compound **5** was successfully purified via HPLC; however, numerous attempts at purifying compound **6** were unsuccessful as **6**, an isomer of **5**, co-eluted with compound **5**. The structure of compound **6** was still resolved, albeit as a mixture with **5**. The ^1^H NMR spectra of compounds **5** and **6** (Figures S28 and S35, respectively) closely resembled that of compound **4** (Figure S17) except for the absence of the shielded methylene cluster between δ_H_ 2.5 and 3.0 and the emergence of extra deshielded signals within the region of ~ δ_H_ 4.2. Of interest in the ^1^H NMR spectrum of **5** (Figure S28) was the peak splitting of the coupled olefinic doublets at δ_H_ 5.92 (*J* = 5.5 Hz) and δ_H_ 6.13 (*J* = 5.5 Hz) vs. that of the broad signals observed for **4** (Figure 4). The ^1^H NMR data (Table 2) of these isomers, particularly the additional resonances at ~ δ_H_ 4.5, implied a structure like that of **4**, save for the substitution of the methylene group at C2. The carbon chemical shift at C2 of ~ δ_C_ 85 (Figure S36) implied a hydroxyl moiety, and compound **6** is analogous to a hydroxy cuparane isolated by Mann (2008) from *Laurencia flexuosa* [31].

The HRGCEIMS spectrum (Figure S34) of compound **5** showed the same molecular formula (C_15_H_19_^79^BrO_2_) as compound **6** with [M-OH] at *m/z* 293.0533 (calculated for 293.0541). Both **5** and **6** possess the same basic carbon skeleton; however, several differences in ^1^H and ^13^C spectral data between the two compounds at C2, C4, C12, C13, and C14 required further investigation. Careful analysis of the NOESY NMR data revealed compound **5** to be a diastereomer of **6** at C1 due to NOESY cross-peaks (Figure S33) from the hydroxy methine H2 (δ_H_ 4.50) to the methyl singlets H12 (δ_H_ 1.38) and H13 (δ_H_ 1.19) in **5**, in contrast to **6*,*** where a correlation only from H2 to H14 was observed (Figure S40).

**Figure 5:**
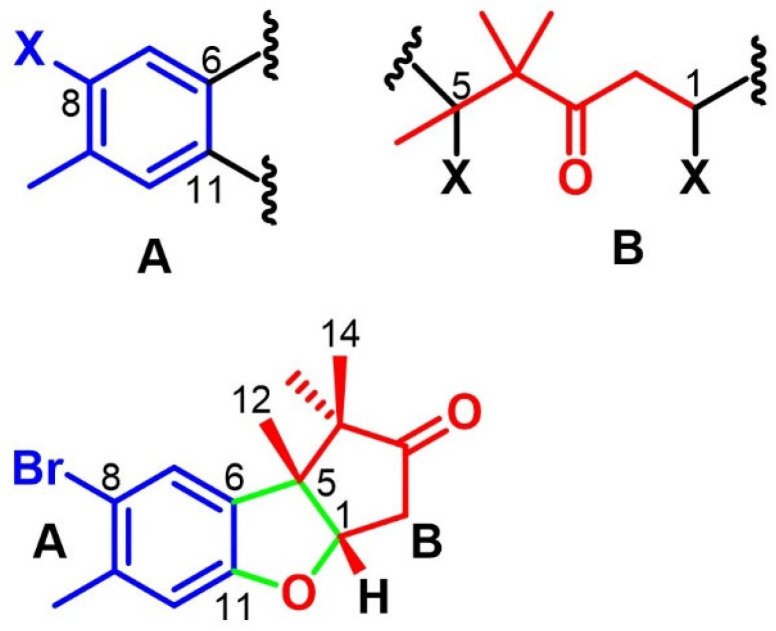
Partial substructures A and B followed by the elucidation of compound **5** with sub-structures A and B attached via a furan ring.

Compound **7** was recrystallized in hexane to afford white, needle-like crystals. Analysis of the HRGCEIMS spectrum (Figure S47) showed isotopic molecular ion signals at *m/z* 308.0356 (calc. 308.0412) and 310.0346. After identifying a total of 15 carbon signals by analysis of the ^13^C NMR (Figure S42) and HMBC (Figure S45) spectra, the molecular formula of **7** was determined to be C_15_H_17_^79^BrO_2_ with a calculated double bond equivalent of seven. The ^13^C NMR spectrum (Figure S42) displayed a deshielded methine signal at δ_C_ 89.1 as well as a cluster of methyl peaks between δ_C_ 20 and 24, like that observed for compound **4**. The ^1^H NMR spectrum of **7** (Figure S41) showed deshielded singlets corresponding to the same tetra-substituted aromatic ring, as previously seen with compound **4**. The appearance of a doublet of doublets at δ_H_ 4.68 (*J* = 6.9, 3.5 Hz) and a methylene multiplet at δ_H_ 2.82 deviated from that obtained for **4**. Following careful analysis of the 1D and 2D NMR data (Table 3), partial structures A and B (Figure 5) were considered. The aromatic substructure A was like that of **4** except for a more highly deshielded quaternary carbon at C11 (δ_C_ 159 vs. δ_C_ 154 for **4**). For substructure B, COSY NMR data (Figure S43) allowed for the construction of a (-CHXCH_2_-) spin system (C1 and C2), while the presence of the carbonyl moiety at C3 was supported by HMBC correlations from the methylene functionality at C2, as well as gem-dimethyl moieties on C4 (Figure S45, Table 3). Additionally, IR spectra revealed a C=O stretch at 1742 cm^−1^ which is typical of five-membered cyclic ketones. The merging of substructures A and B was achieved by HMBC correlations between H12-C6 and H7-C5 (Table 3).

Since the molecular formula revealed the presence of two oxygen atoms and considering a carbon chemical shift at C1 (δ_C_ 89.1), substructure B is proposed to cyclize and form a cyclopentanyl moiety linked to substructure A via a dihydrofuran-like heterocycle as shown in Figure 5. In addition, the aromatic ring and carbonyl moiety within **7** account for a total of five degrees of unsaturation. The additional two double bond equivalents required by the molecular formula must therefore be accommodated by two additional rings within **5**, i.e., the dihydrofuran ring and cyclopentanyl ring. The relative configurations at C1, C4, and C5 were assigned by analysis of NOESY correlations from H1 to H12 and H14 (Table 3 and Figure S46).

The only major spin system (-CH-CH-) differentiating the ^1^H NMR spectrum of compound **8** (Figure S48) compared to **7** (Figure S41) was a doublet at δ_H_ 5.82 (*J* = 6.2 Hz) coupled to a doublet of doublets at δ_H_ 5.62 (*J* = 6.2, 1.9 Hz). Since **8** lacked a shielded methylene multiplet in the ^1^H NMR spectrum (Figure S48, Table S6) and a deshielded carbonyl correlation in the HMBC spectrum, two deshielded carbon signals were detected at δ_C_ 124.8 and δ_C_ 147.7, suggesting the presence of an olefin between C2 and C3 as opposed to a carbonyl at C3 as for compound **7** (Table 3). Compound **8** (Table S6) was isolated previously from *L. flexuosa* by Mann (2008) [31].

**Figure 6:**
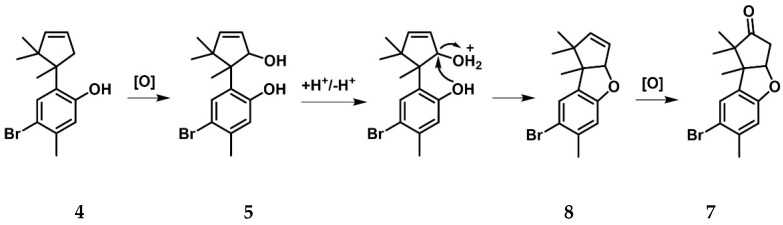
Proposed biosynthetic pathway of compounds **4**, **5**, **8**, and **7**.

Compounds **4**, **5**, **7**, and **8** appear to be part of a proposed biosynthetic series (Figure 6) with compound **4** serving as a plausible precursor. Conversely, the removal of water on the five-membered ring of **5** is likely to give rise to **4**.

### 2.4. Antimicrobial Study

The antibacterial activity of South African marine algal extracts has been assessed [32,33]. However, comparison of results from various studies is difficult due to the different extraction, storage, and testing procedures used [34]. In this study, we investigated the antimicrobial effects of the pure compounds (**1**–**7**) isolated from *L. corymbosa* against a panel of five clinically important pathogens including the Gram-negative *Acinetobacter baumannii* and *Escherichia coli*, the Gram-positive *Enterococcus faecalis* and *Staphylococcus aureus* subsp. *aureus*, and *Candida albicans*. The minimum inhibitory concentrations (MIC) in μg/mL, disk diffusion in mm^2^, and the bioautography results are presented in Table 4. Compound **8** was not tested due to the paucity of the available material. The compounds and the crude extract were initially screened using disk diffusion assays and bioautography with vancomycin, ampicillin, and actidione as controls. Several compounds exhibited activity against *S. aureus* with compounds **1**, **2**, **4**, and **6** as well as the crude extract revealing disk diffusion zones of 18.85, 30.63, 76.18, 24.74, and 31.42 mm^2^, respectively (Table 4). Compound **4** exhibited excellent activity against *S. aureus* at 50 mg/mL as well as an inhibition zone of 24.74 mm^2^ against *C. albicans*. Several compounds showed regrowth of the pathogens, e.g., compounds **4**, **5**, and **6** as well as the crude extract (Table 4), indicating that an initial inhibition of growth took place but that insufficient amounts of the compound were present to kill the organisms. All compounds tested were active against one or more of the pathogens. Compounds **4** and **5** revealed excellent activity against the Gram-negative *A. baumannii* with MICs at 1 μg/mL (Table 4). Compounds **1**–**4** and **7** all showed good activity with MICs at 100 μg/mL against the vancomycin-resistant *E. faecalis*, with compounds **1** and **3** showing potent activity at 1 μg/mL. Compounds **1**, **4a**, **7** and the crude extract also showed good activity with MICs at 100 μg/mL against the Gram-negative *E. coli*. *Candida albicans* was resistant to all the test compounds when tested under liquid media conditions (Table 4). Coprinol, a cuparane isolated from the *Coprinus* fungi, showed moderate activity against Gram-positive multiresistant strains such as penicillin-resistant pneumococci, methicillin- and quinolone-resistant staphylococci, and a vancomycin-intermediate-resistant staphylococcus, with MICs in the range of 6–25 μg/mL [34]. The authors also found no activity against *C. albicans*. The cuparane 10-hydroxycuparaldehyde isolated from *L. obtusa* lamouroux showed good inhibitory activity against *E. coli*, *K. pneumoniae*, *P. mirabilis*, *P. aeruginosa*, *E. faecalis*, and *S. aureus* with MIC (minimum inhibitory concentration) values in the range of 0.08–0.15 mM [35]. Debromolaurinterol and α-bromocuparane isolated from a Bornean *L. snapeyi* showed good antibacterial activity against *S. typhi*, with an MIC/MBC ratio of 2.79 and 2.72, respectively, which is indicative of bactericidal antibiosis [35].

The discrepancies among the different methods of testing for antimicrobial activity may be due to the diffusability of the compounds in and on solid media vs. liquid media. Disk diffusion assays and bioautography are good initial screening tools, whereas true MIC values can be determined in liquid media assays.

## 3. Conclusions

In this, the first phycochemical investigation of a South African *Laurencia corymbosa* J. Agardh, three new (a tricyclic keto-cuparane (**7**) and cuparanes (**4, 5**)) and five known (acetogenins, halo-chamigranes and additional cuparanes) secondary metabolites were isolated. This information can thus be used to assist in future chemotaxonomic investigations of these cryptic species. Marine algae are known to be excellent sources of antimicrobial compounds. Thus, the compounds were screened against several Gram-negative and Gram-positive strains with compounds **4** and **5** exhibiting an MIC of 1 µg/mL against the Gram-negative *A. baumanii* strain. In addition, compounds **1** and **3** showed potent activity at 1 µg/mL against the vancomycin-resistant *E. faecalis*. The bioactivity observed against drug-resistant strains such as *E. faecalis* and *S. aureus* subsp. *aureus* (methicillin resistant), highlights the importance of screening marine algae for compounds which have potential application against these pathogens.

## 4. Experimental Section

### 4.1. General Experimental Procedures

NMR experiments were performed on a Bruker^®^ Avance 600 MHz spectrometer (Rheinstetten, Germany) using standard pulse sequences with CDCl_3_ as the solvent. All spectra were referenced according to residual proton residues in deuterated solvent (CHCl_3_ δ_H_ 7.26, CDCl_3_ δ_C_ 77.0). High-resolution gas chromatography electron impact mass spectrometry (HRGCEIMS) spectra were obtained using a Waters^®^ GCT spectrometer (Milford, MA, USA) in positive mode using an oven ramp from 100 to 280 °C using an HP5 column, helium as the carrier gas (1 mL/min), and an injector temperature of 280 °C. HRESIMS (high-resolution electron spray ionisation mass spectrometry) spectra were obtained using a Waters^®^ Synapt G2 spectrometer (Milford, MA, USA). The ionisation source varied between ESI^+^ and ESI^−^ depending on the compound analyzed, with a cone voltage of 15 V. The lock mass was set with Leucine enkephalin. Both sets of spectra were acquired at the Central Analytical Facility, University of Stellenbosch (Stellenbosch, South Africa). All solvents used were of chromatography grade (LiChrosolv^®^), obtained from Merck^®^ (Darmstadt, Germany), while column chromatography was performed using Merck^®^ Silica gel 60 (0.040–0.063 mm), (Darmstadt, Germany). HPLC was performed using a semi-preparative normal phase Whatman Partisil^®^ 10 M9/50 (9.5 mm × 500 mm) column and a Rheodyne^®^ manual injector containing a Waters^®^ HPLC pump (Milford, MA, USA) with a Spectra physics^®^ RI detector attached to a Ridenki^®^ chart recorder.

### 4.2. Plant Material and Taxonomy

*Laurencia corymbosa* was collected by hand at Noordhoek beach in the south-east coastal region of South Africa in August 2013. All voucher specimens are kept in the seaweed sample repository at the School of Pharmacy, University of the Western Cape. Specimens from these collections were included in detailed taxonomic studies of the genus *Laurencia* in South Africa [36,37]. Though initially described in 1842, little was known about the species until it was demonstrated to be molecularly and morphologically distinct [37]. Although first described in South Africa, this species name has been used in various other world regions, including South-East Asia and the tropical Western Atlantic. Species names of seaweeds are often used erroneously outside of their regions of origin [38], and it seems likely that many of the latter records are misidentifications.

### 4.3. Extraction and Isolation

The algal material, with a dry mass of 41.6 g, was initially steeped in MeOH at room temperature for one hour, after which it was extracted three times with CH_2_Cl_2_-MeOH (2:1, 770 mL × 3) at a constant temperature of 40 °C. The quantity of solvent varied to completely submerge the algal material. The combined organic phases were collected (after an adequate amount of water was added for phase separation) and concentrated under reduced pressure to produce the crude extract (1.17 g, 3.9% dry wt).

Fractionation of the crude was further carried out via step gradient column chromatography using hexane-EtOAc (50 mL, per fraction) as an eluent to yield eight fractions (Scheme S1): A = 100% hexane; B = 9:1 hex-EtOAc, C = 8:2 hex-EtOAc, D = 6:4 hex-EtOAc, E = 4:6 hex-EtOAc, F = 2:8 hex-EtOAc, G = 100% EtOAc, H = 1:1 MeOH-EtOAc). Semi-preparative normal phase HPLC of fr A using hexane as the mobile phase resulted in the isolation of compound **3** while HPLC of fr B using EtOAc-hexane (1:19) as the mobile phase gave compounds **1**, **4**, and **7**. Repeated column chromatography followed by normal phase HPLC using hexane-EtOAc (3:2 and 7:3) as the mobile phase allowed the purification of compounds **2**, **5**, **6**, and **8**.

#### Compounds Isolated from *L. corymbosa*

3-chloro-4,10-dibromo-7,8 epoxychamigrane (**1**): White plate-like crystals, 8 mg (yield 0.027%); ^1^H and ^13^C-NMR (CDCl_3_) data available in Table 5. As previously reported by Howard and Fenical (1975) [19].

Cleavage of the epoxy group in compound **1** to produce compounds **1a** and **1b**: Compound **1** (15 mg) was dissolved in dry Et_2_O (15 mL). In a separate round-bottomed flask, BF_3_·Et_2_O (2 mL) was added to Et_2_O (15 mL) and cooled to −70 °C in a dry ice/acetone bath. The previously prepared solution of compound **1** was gradually added to the BF_3_·Et_2_O solution, after which stirring occurred for 18 h under a N_2_ stream, allowing the vessel to slowly reach room temperature. The reaction mixture was quenched with NaHCO_3_ (30 mL), filtered, and dried under vacuum. The resultant crude mixture was purified via normal phase HPLC (4:1 Hex: EtOAc) yielding pure compounds **1a** (6.1 mg, 40.6%) and **1b** (2.3 mg, 15.3%).

3-chloro-9-hydroxy-4,10-dibromo-7,8-epoxychamigrane (**2**): White crystals, 2 mg (yield 0.007%); ^1^H and ^13^C-NMR (CDCl_3_) data available in Table S3. As previously reported by Elsworth and Thomson (1989) [24].

Neolaurencenyne ((3*Z*,6*Z*,9*Z*)-pentadeca-3,6,9-trien-1-yne) (**3**): 14.1 mg (yield 0.047%); ^1^H and ^13^C-NMR (CDCl_3_) data available in Table S4. As previously isolated by Kigoshi et al. (1981) [25].

4-bromo-5-methyl-2-(1,2,2-trimethylcyclopent-3-en-1-yl)phenol (**4**): Clear oil, 14.5 mg (yield 0.049%); [α]_D_^25^ + 93.2° (c = 0.005, MeOH); ^1^H and ^13^C-NMR (CDCl_3_) data available in Table 5; IR *V*_max_ 3397; 2916; 1375 cm^−1^; HRGCEIMS *m/z* 294.0620 (Calculated for 294.0619); C_15_H_19_^79^BrO.

4-bromo-5-methyl-2-(1,2,2-trimethylcyclopent-3-en-1-yl)phenyl acetate (**4a**): White solid; [α]_D_^25^ + 61.2^o^ (c = 0.002, MeOH); ^1^H and ^13^C-NMR (CDCl_3_) data available in Table S5; IR *V*_max_ 3402; 2962; 2921; 1742 cm^−1^; HRGCEIMS, [M+H]^+^ *m/z* 337.0789 (calculated for 337.0803); C_17_H_21_^79^BrO_2_. Compound **4** (4 mg) was weighed out and placed in a round-bottomed flask with 10 drops of pyridine and 20 drops of acetic anhydride. The reaction mixture was left to stir overnight at ambient temperature, after which HCl (1M, 12 mL) was used to acidify the mixture. EtOAc (3 × 10 mL) was used to extract the resultant product, after which semi-preparative TLC was used (4:6 hex:EtOAc) to obtain the acetate derivative compound **4a**.

4-bromo-2-[(1S*,5S*)-5-hydroxy-1,2,2-trimethylcyclopent-3-en-1-yl]-5-methylphenol (**5**): Clear oil, 4 mg (yield 0.013%); ^1^H and ^13^C-NMR (CDCl_3_) data available in Table 2; IR *V*_max_ 3296; 2962; 2151; 1390 cm^−1^; HRGCEIMS [M-OH] *m/z* 293.0533 (calculated for 293.0541); C_15_H_19_^79^BrO_2_.

8-bromo-6-[(1R*,5S*)-2-hydroxy-1,5,5-trimethylcyclopent-3-en-1-yl]-9-methylphenol (**6**): Colourless oil, 6 mg (yield 0.020%); ^1^H and ^13^C NMR (CDCl_3_) data available in Table 2. As previously reported by Mann (2008) [31].

(3aS*,8bR*)-7-bromo-1,1,6,8b-tetramethyl-1,3,3a,8b-tetrahydro-2*H*-benzo[b]cyclopenta[d]furan-2-one (**7**): White crystals, 19.8 mg (yield 0.066%); [α]_D_^25^ − 86.5° (c = 0.008, MeOH); ^1^H and ^13^C-NMR (CDCl_3_) data available in Table 3; IR *V*_max_ 2972, 1742, 1471, 1392 cm^−1^; HRGCEIMS *m/z* 308.0356 (calculated for 308.0412); C_15_H_17_^79^BrO_2_.

7-bromo-1,1,6,8b-tetramethyl-3a,8b-dihydro-1*H*-benzo[b]cyclo-penta[d] furan (**8**): Colourless oil, 3 mg (yield 0.010%); ^1^H and ^13^C-NMR (CDCl_3_) data available in Table S5. As previously reported by Mann (2008) [31].

### 4.4. Antimicrobial Activity Study

#### 4.4.1. Preparation of the Extracts

The seaweed extract was re-dissolved in 1 mL ethyl acetate to a final concentration of 1 mg/mL.

#### 4.4.2. Initial Antimicrobial Screening—Disk Diffusion Assays

Filter disks were sterilised by autoclaving and were impregnated with a 20 µg solution of the extract (20 µL of a 1 mg/mL solution per disk). Ten filter disks were prepared per sample tested. The filter disks were allowed to dry overnight. Control filter disks containing no additive, containing ethyl acetate, and containing ampicillin/vancomycin/actidione were used to ensure that the killing of the test strains was due to the extracts and not the organic solvent, that the filter disks were sterile, and that the strains could be killed when a standard antimicrobial was used.

#### 4.4.3. Preparation of the Test Strains

The following strains were used to determine the antimicrobial activity of the extracts: *Acinetobacter baumannii* ATCC BAA-1605 (tryptic soy broth, TSB; Merck Millipore, MA, USA), *Enterococcus faecalis* ATCC 51299 (brain heart infusion broth supplemented with 4 μg/mL vancomycin; Merck Millipore), *Escherichia coli* ATCC 25922 (TSB), *Staphylococcus aureus* subsp. *aureus* ATCC 33591 (TSB), and *Candida albicans* ATCC 24433 (nutrient broth; Merck Millipore, Burlington. MA, USA). The strains were inoculated into 5 mL of the growth media indicated (prepared according to the manufacturer’s instructions) and incubated at 37 °C for 24 h. The optical density (OD) of the cultures was measured at 600 nm and the culture OD adjusted to 0.5 using sterile growth media prior to use in the disk diffusion assays. A measure of 100 µL of the culture was spread-plated onto agar media (the same as the growth media, but with 15 g/L bacteriological agar added) and the filter disks were placed on the agar plate prior to incubation at 37 °C for 24 h. Duplicate filters per sample were used. After 24 h of growth, the plates were examined for the presence of zones of inhibition.

#### 4.4.4. Bioautography

The same test strains were used in the bioautography experiment. The same amount of sample was spotted in duplicate onto aluminum-backed silica gel 60 F_254_ thin layer chromatography (TLC) plates (Merck, Burlington, MA, USA) and allowed to dry. Sterile camera lens tissues were saturated with the test strains, and the tissue was placed over the TLC plate to allow for the transfer of the test strain. The TLC plates were placed in a plastic container containing moist paper towel and incubated at 37 °C for 24 h. The TLC plates were treated with the tetrazolium salt, i.e., 3-(4,5-dimethylthiazol-2-yl)-2,5-diphenyltetrazolium bromide (MTT) (0.25%, w/v in phosphate-buffered saline, PBS), re-incubated and evaluated for the appearance of purple spots (indicative of growth) or white spots (indicative of killing).

#### 4.4.5. Liquid Assays

Measures of 400 µL of the 1 mg/mL solutions were transferred to new tubes and allowed to evaporate. The samples were resuspended in 1 mL DMSO to give a final concentration of 400 µg/mL for each sample. To determine the minimal inhibitory concentration (MIC) of the samples, several dilutions were prepared using 0.45–45 µL of sample in DMSO and made up to 180 µL with the culture to give final concentrations of 1–100 µg/mL. Vancomycin, ampicillin, or actidione was used as the positive control, while the sterile control simply contained distilled water, broth, and 50 µL of DMSO. The test strains were grown in optimal growth media as indicated previously. After 24 h of growth, the OD_600_ of the culture was determined and adjusted to 0.8 for the purpose of this experiment. The samples and cultures were dispensed into microtiter plates and incubated for 24 h at 37 °C. A measure of 20 µL of 0.25% (*w*/*v*) MTT in PBS was added to each well, and the plates were incubated at 37 °C for 3 h. A measure of 100 µL DMSO was added to each well and incubated at room temperature for 4 h. The OD of the samples was determined at 570 nm. The MIC was determined to be where all colour is lost as compared to the control containing the DMSO equivalent.

## Figures and Tables

**Table 1:** NMR spectroscopic data for compound **4**.

Atom No.	δ_C_ (Mult.)	δ_H_, Mult, *J* (Hz)	COSY	HMBC
1	56.3 (C)	-	-	-
2a	47.6 (CH_2_)	3.01, d, 16.1	H2b	-
2b		2.50, d, 16.1	H2a	-
3	126.8 (CH)	5.83, br s	H2a, H2b	C1 ^1^
4	144.3 (CH)	5.67, br s	H2a, H2b ^1^	-
5	49.9 (C)	-	-	-
6	133.4 (C)	-	-	-
7	132.0 (CH)	7.41, s	H12, H15	C5, C8, C9, C11, C15
8	114.7 (C)	-	-	-
9	136.5 (C)	-	-	-
10	119.0 (CH)	6.65, s	H15	C6, C8, C11, C15
11	154.1 (C)	-	-	-
12	25.3 (CH_3_)	1.47, s	-	C1, C2, C5, C6
13	25.9 (CH_3_)	1.19, s	-	C4^1^, C5, C14
14	24.9 (CH_3_)	0.75, s	-	C4, C5, C13
15	22.1 (CH_3_)	2.30, s	H7, H10	C8, C9, C10

^1^ Indicates a weak correlation. Abbreviations: Mult.—multiplicity; COSY—Correlation Spectroscopy; HMBC—Heteronuclear Multiple Bond Correlation.

**Table 2:** NMR spectroscopic data for compound **6** as a mixture with **5**.

	5			6		
Atom No.	δ_C_	δ_H_, Mult, *J* (Hz)	NOESY	δ_C_	δ_H_, Mult, *J* (Hz)	NOESY
1	55.2	-	-	56.0	-	-
2	85.3	4.50, d, 7.4	H12, H13	85.6	4.28, br s	H14
3	131.8	5.92, d, 5.5	H4	131.0	6.02, d, 5.5	H4
4	141.4	6.13, d, 5.5	H3	144.6	6.33, d, 5.5	H3
5	48.9	-	-	47.1	-	-
6	128.7	-	-	129.7	-	-
7	132.2	7.27, s	H12	132.1	7.26, s	H12
8	115.5	-	-	115.1	-	-
9	137.3	-	-	137.0	-	-
10	119.7	6.64, s	H15	119.0	6.62, s	-
11	154.0	-	-	153.8	-	-
12	23.4	1.38, s	H2, H7, H13	26.4	1.49, s	H7, H13
13	23.4	1.19, br s	H2, H12, H14	18.8	1.22, s	H12, H14
14	20.3	0.66, s	H13	27.1	0.66, s	H2, H13
15	22.2	2.30, s	H10	22.3	2.29, s	H10

Abbreviations: Mult.—multiplicity; NOESY—Nuclear Overhauser Effect Spectroscopy.

**Table 3:** NMR spectroscopic data for compound **7**.

Atom No.	δ_C_ (Mult.)	δ_H_, Mult, *J* (Hz)	COSY	HMBC	NOESY
1	89.1 (CH)	4.86, dd, 6.9, 3.5	H2	-	H2, H12, H14
2	40.7 (CH_2_)	2.82, m	H1	C3	H12, H13
3	218.5 (C)	-	-	-	-
4	51.9 (C)	-	-	-	-
5	55.2 (C)	-	-	-	-
6	132.1 (C)	-	-	-	-
7	127.6 (CH)	7.23, s	-	C9, C11	H12, H13, H14
8	115.4 (C)	-	-	-	-
9	138.3 (C)	-	-	-	-
10	112.4 (CH)	6.69, s	H15	C15	H15
11	159.2 (C)	-	-	-	-
12	21.8 (CH_3_)	1.32, s	-	C1, C4, C5, C6	H1, H2, H7
13	23.1 (CH_3_)	0.86, s	H1, H2	C3, C4, C5, C14	H2, H7
14	20.2 (CH_3_)	1.08, s	-	C3, C4, C5, C13	H1
15	23.3 (CH_3_)	2.34, s	-	C8, C9, C10	-

Abbreviations: Mult.—multiplicity; COSY—Correlation Spectroscopy; HMBC—Heteronuclear Multiple Bond Correlation; NOESY—Nuclear Overhauser Effect Spectroscopy.

**Table 4:** Antimicrobial data for the crude extract and the compounds isolated. MIC values are given in μg/mL, while the disk diffusion (in mm^2^) and bioautography results are also shown.

	Ab		Ef		Ec		Sa		Ca	
Sample	MIC (μg/mL)	dd (Ba)	MIC (μg/mL)	dd (Ba)	MIC (μg/mL)	dd (Ba)	MIC (μg/mL)	dd (Ba)	MIC (μg/mL)	dd (Ba)
**1**	-	-	1	-	100	-	-	18.85	-	-
**2**	-	-	100	-	-	-	-	30.63	-	-
**3**	-	-	1	-	-	-	-	-	-	-
**4**	1	(rg)	100	(rg)	-	-	50	76.18	-	24.74 (rg)
**4a**	-	-	100	-	100	-	-	-	-	-
**5**	1	-	-	(rg)	-	-	-	(rg)	-	(rg)
**6**	-	-	100	(rg)	-	-	-	24.74 (rg)	-	(rg)
**7**	-	-	-	-	100	-	-	-	-	-
crude	-	-	-	(rg)	100	-	-	31.42	-	-
Vancomycin	100	-	100	-	100	-	20	-	100	-
Ampicillin	100	-	1	-	1	-	>100	-	100	-
Actidione	100	-	50	-	100	-	100	-	>100	-

Ab: *Acinetobacter baumannii* ATCC BAA-1605; Ef: *Enterococcus faecalis* ATCC 51299; Ec: *Escherichia coli* ATCC 25922; Sa: *Staphylococcus aureus* subsp. *aureus* ATCC 33591; Ca: *Candida albicans* ATCC 24433. MIC (μg/mL): minimum inhibitory concentration; dd: Disk diffusion (mm^2^); in parentheses: Ba: Bioautography (rg = with regrowth). -: no activity observed.

**Table 5:** NMR spectroscopic data for compound **1**.

Atom No.	δ_C_ (Mult.)	δ_H_, Mult, *J* (Hz)	COSY	HMBC	NOESY
1a	26.2 (CH_2_)	1.67, dq, 15.2, 5.7	H1b, H2a	-	H1b, H13, H14
1b		1.98, dt, 15.2, 5.7	H1a, H2a, H2b	-	H1a, H13
2a	40.4 (CH_2_)	2.47, dt, 14.6, 5.7	H1a, H1b	C1, C3, C15	H2b, H14
2b		2.38, dd, 14.6, 2.9	H1b	C1, C3, C4, C6, C15	H2a, H15
3	71.1 (C)	-	-	-	-
4	62.1 (CH)	4.77, dd, 13.3, 4.7	H5a, H5b	C3, C5, C15	H5a, H14
5a	39.1 (CH_2_)	2.84, br d, 13.3	H4, H5b	-	H4
5b		2.10, t, 13.3	H4, H5a	C4, C7, C6, C11	H1a, H12, H13, H15
6	44.6 (C)	-	-	-	-
7	62.0 (C)	-	-	-	-
8	62.0 (CH)	2.92, t, 3.0	H9	C9, C10, C14	H9, H14
9	32.7 (CH_2_)	2.61, dd, 7.7, 3.0	H8, H10	C7/8, C10, C11	H8, H10, H12, H13
10	57.6 (CH)	4.06, t, 7.7	H9, H12	C6, C8, C9, C12, C13	H1a, H9, H13
11	42.3 (C)	-	-	-	-
12	21.3 (CH_3_)	1.18, s	H10	C6, C11, C13	H5a, H5b, H9
13	27.3 (CH_3_)	1.08, s	-	C6, C11, C2	H1a, H1b, H5b, H15
14	25.8 (CH_3_)	1.50, s	-	C6, C7/8	H2a, H4, H8
15	24.0 (CH_3_)	1.74, s	-	C2, C3, C4	H1a, H2b, H5b, H13

Abbreviations: Mult.—multiplicity; COSY—COrrelation SpectroscopY; HMBC—Heteronuclear Multiple Bond Correlation NOESY—Nuclear Overhauser Effect SpectroscopY.

## Data Availability

Data are contained within the article or supplementary material. The data presented in this study are available in the paper and supplementary material of this paper.

## Supplementary Materials

The following supporting information can be downloaded at: https://www.mdpi.com/article/10.3390/molecules28052063/s1, Figure S1. Images of *Laurencia corymbosa* (Francis, 2014) (a) habit, (b) cross section through the thallus with the outermost cortical cells and spaces between medullary and cortical cells in view and (c) cortical cells showing one corps en cerise per cell (40x magnification); Figure S2: ^1^H NMR spectrum (CDCl_3_, 600 MHz) of compound **1**; Figure S3: ^13^C NMR spectrum (CDCl_3_, 150 MHz) of compound **1**; Figure S4: ^1^H-^1^H COSY correlations of compound **1**; Figure S5: Partial multiplicity-edited HSQC spectrum of compound **1** showing methylene correlations; Figure S6: Partial HMBC spectrum of compound **1** showing key correlations; Figure S7: Partial HMBC spectrum of compound **1** showing key correlations; Figure S8: NOESY NMR spectrum (CDCl_3_, 600 MHz) of compound **1**; Figure S9: ^1^H NMR spectrum (CDCl_3_, 600 MHz) of compound **1a**; Figure S10: ^1^H NMR spectrum (CDCl_3_, 600 MHz) of compound **1b**; Figure S11: ^13^C NMR spectrum (CDCl_3_, 150 MHz) of compound **1b**; Figure S12: ^1^H NMR spectrum (CDCl_3_, 600 MHz) of compound **2**; Figure S13: ^13^C NMR spectrum (CDCl_3_, 600 MHz) of compound **2**; Figure S14: ^1^H NMR spectrum (CDCl_3_, 600 MHz) of compound **3**; Figure S15: ^13^C NMR spectrum (CDCl_3_, 150 MHz) of compound **3**; Figure S16: NOESY NMR spectrum (CDCl_3_, 600 MHz) of compound **3**; Figure S17: ^1^H NMR spectrum (CDCl_3_, 600 MHz) of compound **4**; Figure S18: ^13^C NMR spectrum (CDCl_3_, 150 MHz) of compound **4**; Figure S19: COSY NMR spectrum (CDCl_3_, 600 MHz) of compound **4**; Figure S20: HSQC NMR spectrum (CDCl_3_, 600 MHz) of compound **4**; Figure S21: HMBC NMR spectrum (CDCl_3_, 600 MHz) of compound **4**; Figure S22: Partial HMBC spectrum of compound **4** showing key correlations; Figure S23: NOESY NMR spectrum (CDCl_3_, 600 MHz) of compound **4**; Figure S24: HRGCEIMS spectra of compound **4**; Figure S25: ^1^H NMR spectrum (CDCl_3_, 600 MHz) of compound **4a**; Figure S26: ^13^C NMR spectrum (CDCl_3_, 150 MHz) of compound **4a**; Figure S27: Expansion of the HRESIMS spectrum of compound **4a**; Figure S28: ^1^H NMR spectrum (CDCl_3_, 600 MHz) of compound **5**; Figure S29: ^13^C NMR spectrum (CDCl_3_, 150 MHz) of compound **5**; Figure S30: COSY NMR spectrum (CDCl_3_, 600 MHz) of compound **5**; Figure S31: HSQC NMR spectrum (CDCl_3_, 600 MHz) of compound **5**; Figure S32: HMBC NMR spectrum (CDCl_3_, 600 MHz) of compound **5**; Figure S33: NOESY NMR spectrum (CDCl_3_, 600 MHz) and key NOESY correlations for compound **5**; Figure S34: HRGCEIMS spectrum of compound **5**; Figure S35: ^1^H NMR spectrum (CDCl_3_, 600 MHz) of compound **6**; Figure S36: ^1^H NMR spectrum (CDCl_3_, 600 MHz) of compound **7**; Figure S37: ^13^C NMR spectrum (CDCl_3_, 150 MHz) of compound **7**; Figure S38: COSY NMR spectrum (CDCl_3_, 600 MHz) of compound **7**; Figure S39: HSQC NMR spectrum (CDCl_3_, 600 MHz) of compound **7**; Figure S40: HMBC NMR spectrum (CDCl_3_, 600 MHz) of compound **7**; Figure S41: NOESY NMR spectrum (CDCl_3_, 600 MHz) of compound **7**; Figure S42: HRESIMS spectrum of compound **7**; Figure S43: ^1^H NMR spectrum (CDCl_3_, 600 MHz) of compound **8**. Compound **8** was isolated as a mixture with compound **7**; Figure S44: ^13^C NMR spectrum (CDCl_3_, 150 MHz) of compound **8**; Figure S45: COSY NMR spectrum (CDCl_3_, 600 MHz) of compound **8**; Figure S46: HSQC NMR spectrum (CDCl_3_, 600 MHz) of compound **8**; Figure S47: HMBC NMR spectrum (CDCl_3_, 600 MHz) of compound **8**; Figure S48: NOESY NMR spectrum (CDCl_3_, 600 MHz) of compound **8**; Table S1: NMR spectroscopic data of compound **1a**; Table S2: NMR spectroscopic data of compound **1b**; Table S3: NMR spectroscopic data of compound **2**; Table S4: NMR spectroscopic data of compound **4a**; Table S5: NMR spectroscopic data of compound **6**; Table S6. NMR spectroscopic data of compound; Scheme S1. Isolation of compounds **1**–**8** from *L. corymbosa.*
