# Supplementary material for: Antimicrobial Activity of the Secondary Metabolites Isolated from a South African Red Seaweed, Laurencia corymbosa
Source: Molecules. 2023 Feb 22;28(5):2063. doi: 10.3390/molecules28052063 (PMC10003847; doi:10.3390/molecules28052063)
Supplement: Supplementary file 1 [file molecules-28-02063-s001.zip › molecules-2206596-supplementary.pdf]

molecules

MDPI

Supplementary information

Antimicrobial activity of the secondary metabolites isolated from a South African *Laurencia corymbosa*.

Jameel Fakee[1], John J. Bolton[2], Marilize Le Roes-Hill[3], Kim A. Durrell[3], Edith Antunes[4], and Denzil R. Beukes[5,*]

[1] Faculty of Pharmacy, Rhodes University, Makhanda (Grahamstown), 6140, South Africa
[2] Department of Biological Sciences, University of Cape Town, Rondebosch, 7701, South Africa
[3] Biocatalysis and Technical Biology Research Group, Institute of Biomedical and Microbial Biotechnology, Cape Peninsula University of Technology, Bellville, 7535, South Africa
[4] Department of Chemistry, University of the Western Cape, Bellville, 7535, South Africa
[5] School of Pharmacy, University of the Western Cape, Bellville, 7535, South Africa
* Correspondence: dbeukes@uwc.ac.za; Tel.: +27219592352

Contents

Figure S1. Images of *Laurencia corymbosa* (Francis, 2014) (a) habit, (b) cross section through the thallus with the outermost cortical cells and spaces between medullary and cortical cells in view and (c) cortical cells showing one corps en cerise per cell (40x magnification). 5

Scheme S1. Isolation of compounds 1-8 from *L. corymbosa* 6

Figure S2. ^{1}H NMR spectrum ($CDCl_3$, 600 MHz) of compound 1 7

Figure S3. ^{13}C NMR spectrum ($CDCl_3$, 150 MHz) of compound 1 7

Figure S4. ^{1}H-^{1}H COSY correlations of compound 1 8

Figure S5. Partial multiplicity-edited HSQC spectrum of compound 1 showing methylene correlations 8

Figure S6. Partial HMBC spectrum of compound 1 showing key correlations 8

Figure S7. Partial HMBC spectrum of compound 1 showing key correlations 9

Figure S8. NOESY NMR spectrum ($CDCl_3$, 600 MHz) of compound 1 9

1.2 Compound 1a 10

Table S1. NMR spectroscopic data of compound 1a 10

Figure S9. ^{1}H NMR spectrum ($CDCl_3$, 600 MHz) of compound 1a 10

1.3 Compound 1b 11

Table S2. NMR spectroscopic data of compound 1b 11

Figure S10. ^{1}H NMR spectrum ($CDCl_3$, 600 MHz) of compound 1b 11

Figure S11. ^{13}C NMR spectrum ($CDCl_3$, 150 MHz) of compound 1b 12

1.4 Compound 2 12

Table S3. NMR spectroscopic data of compound 2 12

Figure S12. ^{1}H NMR spectrum ($CDCl_3$, 600 MHz) of compound 2 13

Figure S13. ^{13}C NMR spectrum ($CDCl_3$, 600 MHz) of compound 2 13

1.5 Compound 3 14

Table S4. NMR spectroscopic data of compound 3 14

Figure S14. ^{1}H NMR spectrum ($CDCl_3$, 600 MHz) of compound 3 14

Figure S15. ^{13}C NMR spectrum ($CDCl_3$, 150 MHz) of compound 3 15

Figure S16. NOESY NMR spectrum ($CDCl_3$, 600 MHz) of compound 3 15

1.4 Compound 4 16

Figure S17. ^{1}H NMR spectrum ($CDCl_3$, 600 MHz) of compound 4 16

Figure S18. ^{13}C NMR spectrum ($CDCl_3$, 150 MHz) of compound 4 16

Figure S19. COSY NMR spectrum ($CDCl_3$, 600 MHz) of compound 4 17

Figure S20. HSQC NMR spectrum ($CDCl_3$, 600 MHz) of compound 4 17
Figure S21. HMBC NMR spectrum ($CDCl_3$, 600 MHz) of compound 4 18
Figure S22. Partial HMBC spectrum of compound 4 showing key correlations 18
Figure S23. NOESY NMR spectrum ($CDCl_3$, 600 MHz) of compound 4 19
Figure S24. HRGCEIMS spectra of compound 4 19
1.5 Compound 4a 20
Table S5. NMR spectroscopic data of compound 4a 20
Figure S25. ^1H NMR spectrum ($CDCl_3$, 600 MHz) of compound 4a 20
Figure S26. ^{13}C NMR spectrum ($CDCl_3$, 150 MHz) of compound 4a 21
Figure S27. Expansion of the HRESIMS spectrum of compound 4a 21
1.6 Compound 5 22
Figure S28. ^1H NMR spectrum ($CDCl_3$, 600 MHz) of compound 5 22
Figure S29. ^{13}C NMR spectrum ($CDCl_3$, 150 MHz) of compound 5 22
Figure S30. COSY NMR spectrum ($CDCl_3$, 600 MHz) of compound 5 23
Figure S31. HSQC NMR spectrum ($CDCl_3$, 600 MHz) of compound 5 23
Figure S32. HMBC NMR spectrum ($CDCl_3$, 600 MHz) of compound 5 24
Figure S33. NOESY NMR spectrum ($CDCl_3$, 600 MHz) of compound 5 25
1.7 Compound 6 26
Figure S35. ^1H NMR spectrum ($CDCl_3$, 600 MHz) of compound 6. Compound 6 was isolated as a mixture with compound 5. 26
Figure S36. ^{13}C NMR spectrum ($CDCl_3$, 150 MHz) of compound 6 27
Figure S37. COSY NMR spectrum ($CDCl_3$, 600 MHz) of compound 6 27
Figure S38. HSQC NMR spectrum ($CDCl_3$, 600 MHz) of compound 6 28
Figure S39. HMBC NMR spectrum ($CDCl_3$, 600 MHz) of compound 6 28
Figure S40. NOESY NMR spectrum ($CDCl_3$, 600 MHz) of compound 6 29
1.8 Compound 7 30
Figure S41. ^1H NMR spectrum ($CDCl_3$, 600 MHz) of compound 7 30
Figure S42. ^{13}C NMR spectrum ($CDCl_3$, 150 MHz) of compound 7 30
Figure S43. COSY NMR spectrum ($CDCl_3$, 600 MHz) of compound 7 31
Figure S44. HSQC NMR spectrum ($CDCl_3$, 600 MHz) of compound 7 31
Figure S45. HMBC NMR spectrum ($CDCl_3$, 600 MHz) of compound 7 32
Figure S46. NOESY NMR spectrum ($CDCl_3$, 600 MHz) and key NOESY correlations for compound 7 32
Figure S47. HRGCEIMS spectrum of compound 7 33

1.9 Compound 8 34

Table S6. NMR spectroscopic data of compound 8 34

Figure S48. ^{1}H NMR spectrum ($CDCl_3$, 600 MHz) of compound 8 34

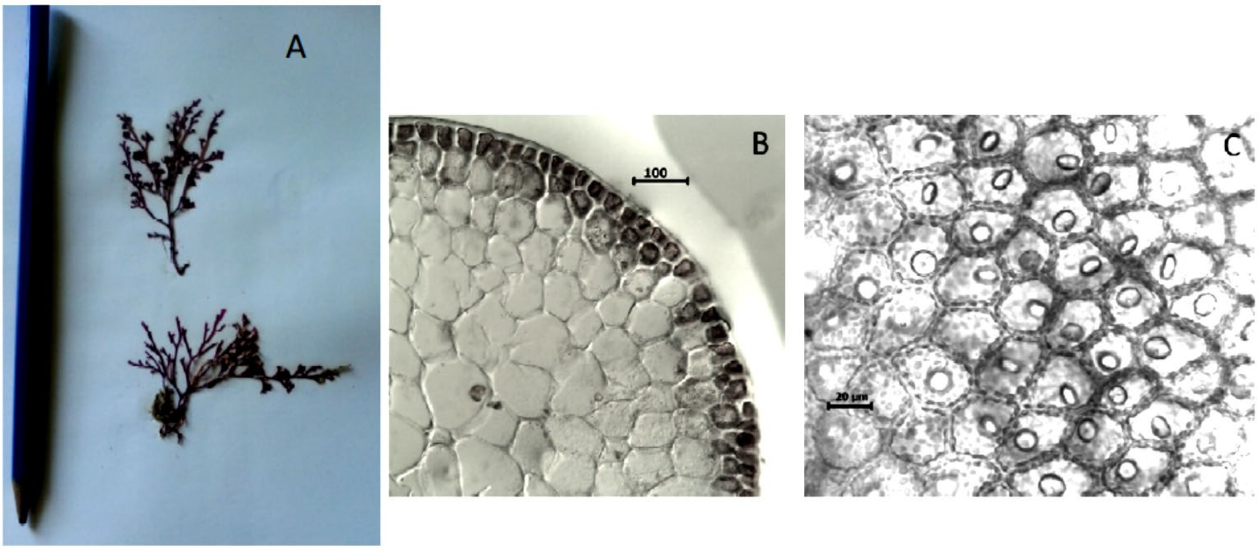

Figure S1. Images of *Laurencia corymbosa* (Francis, 2014) (**a**) habit, (**b**) cross section through the thallus with the outermost cortical cells and spaces between medullary and cortical cells in view and (**c**) cortical cells showing one corps en cerise per cell (40 × magnification).

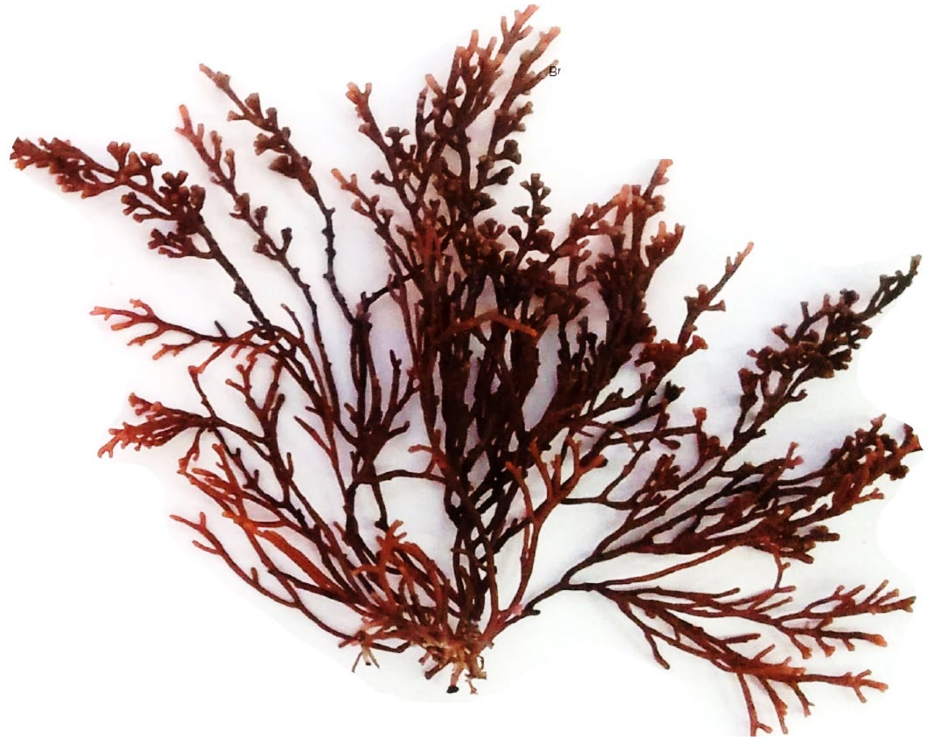

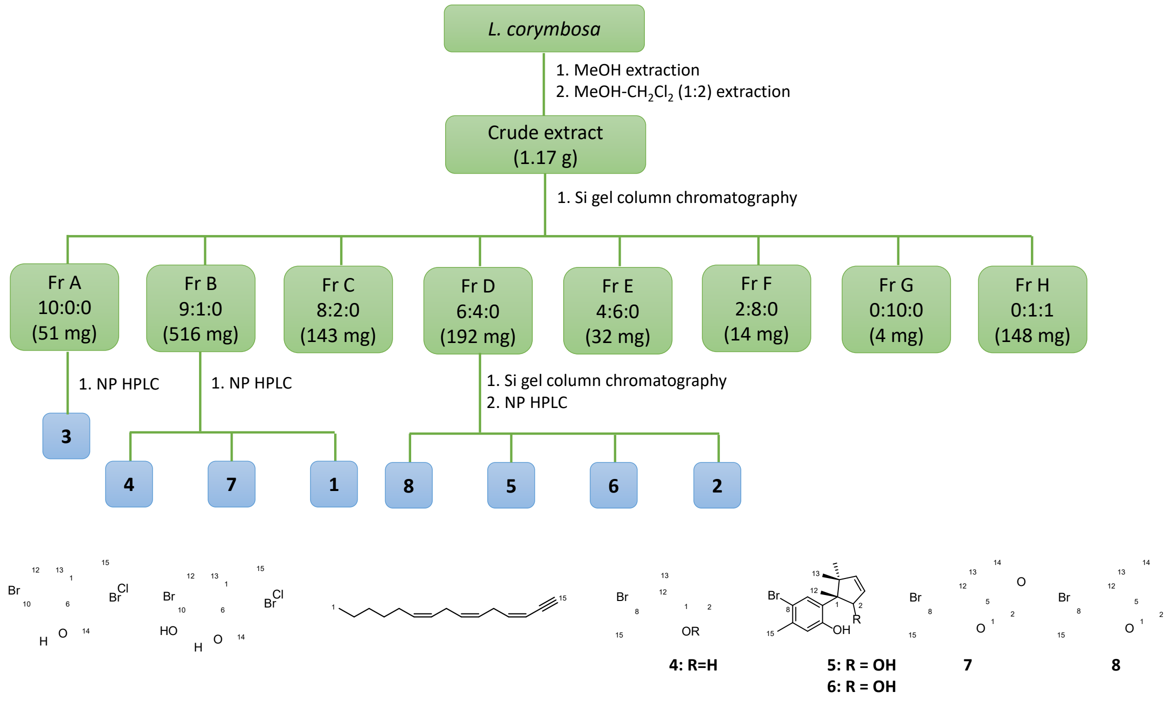

Scheme S1. Isolation of compounds **1-8** from *L. corymbose*.

Spectroscopic data of compounds isolated

1.1. Compound 1

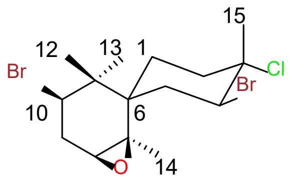

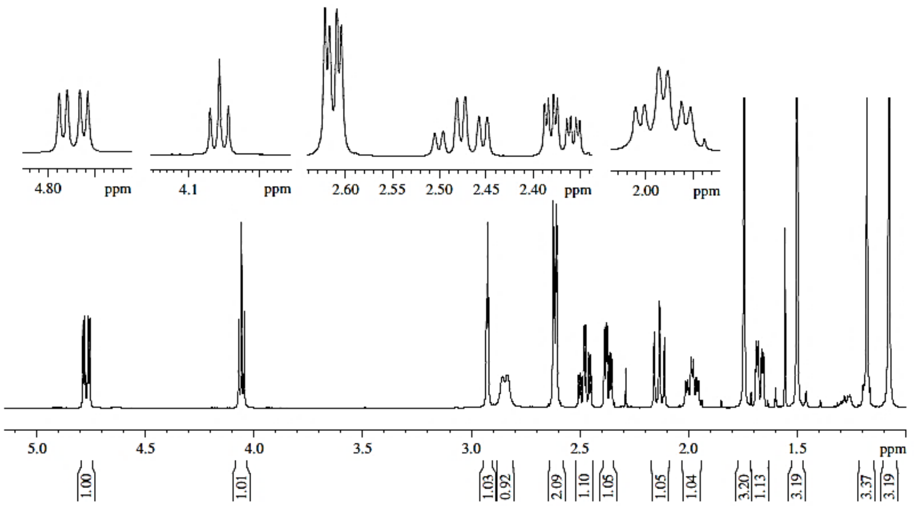

Figure S2. ^{1}H NMR spectrum ($CDCl_3$, 600 MHz) of compound **1**.

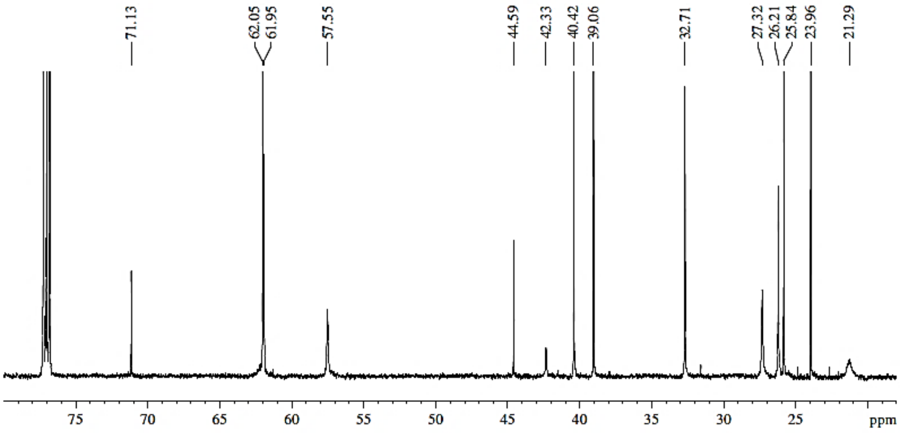

Figure S3. ^{13}C NMR spectrum ($CDCl_3$, 150 MHz) of compound **1**.

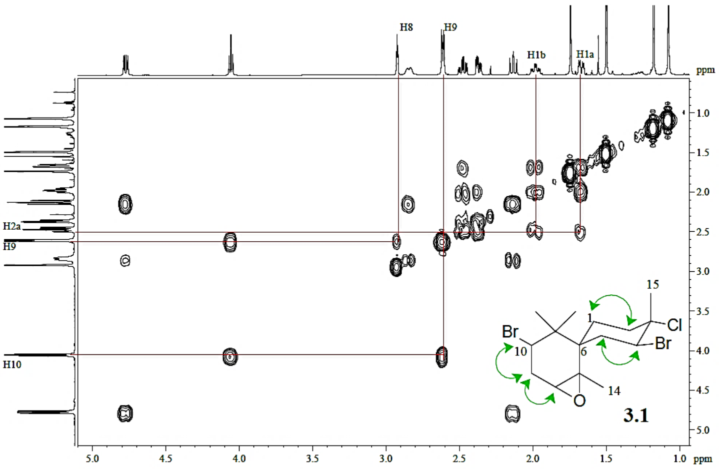

Figure S4. ^{1}H-^{1}H COSY correlations of compound **1**.

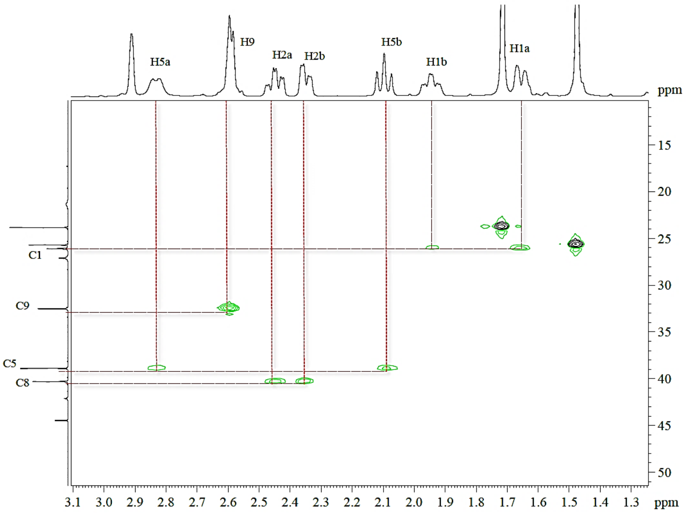

Figure S5. Partial multiplicity-edited HSQC spectrum of compound **1** showing methylene correlations.

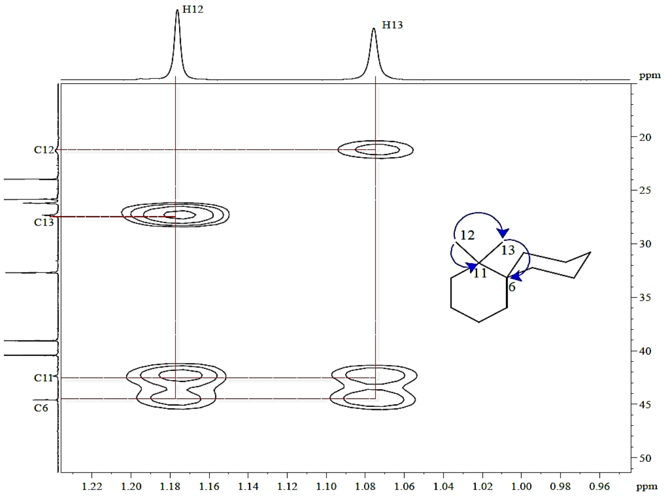

Figure S6. Partial HMBC spectrum of compound **1** showing key correlations.

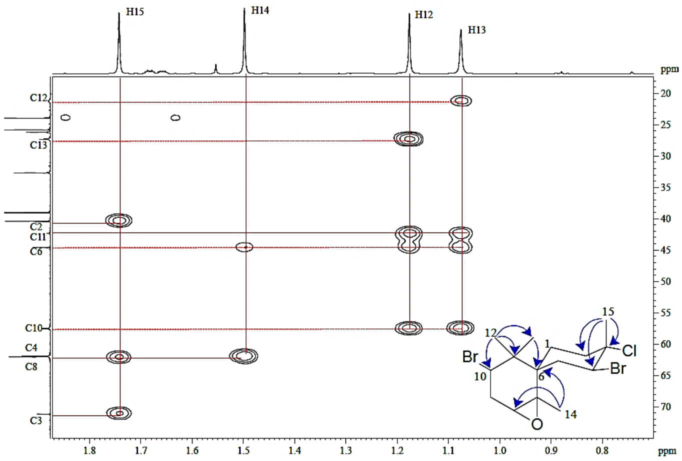

Figure S7. Partial HMBC spectrum of compound **1** showing key correlations.

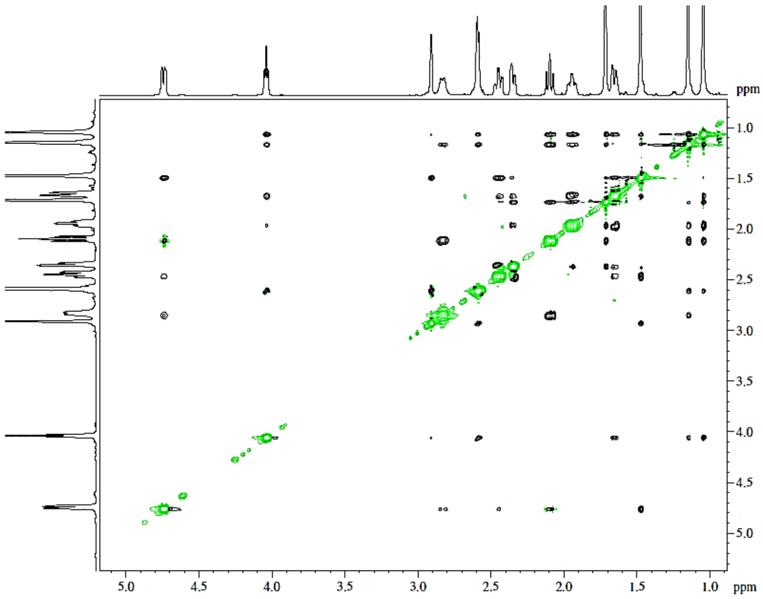

Figure S8. NOESY NMR spectrum ($CDCl_3$, 600 MHz) of compound **1**.

1.2 Compound 1a

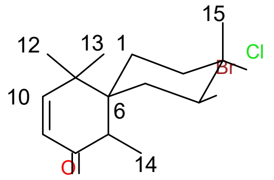

Table S1. NMR spectroscopic data of compound **1a**.

Carbon No	δ_C	δ_C mult	δ_H, mult, *J* (Hz)	COSY	HMBC
1a	28.3	CH_2	1.43, dq, 15.0, 2.9	H2b	-
1b			1.71, m	H2a	-
2a	37.7	CH_2	2.19, td, 14.8, 4.1	H1b	-
2b			2.35, dt, 14.8, 4.1	H1a	-
3	71.8	C	-	-	-
4	59.7	CH	4.45, dd, 13.3, 4.5	H5a, H5b	-
5a	37.1	CH_2	1.98, t, 13.3	H4	-
5b			2.40, td, 13.3, 4.5	H4	-
6	43.1	C	-	-	-
7	44.1	CH	2.90, q, 7.8	H14	C6, C8, C11, C14
8	201.5	C	-	-	-
9	123.8	CH	5.82, d, 10.2	H10	-
10	156.0	CH	6.43, d, 10.2	H9	C8
11	39.1	C	-	-	-
12	25.4	CH_3	1.09, s	-	C6, C10, C11, C13
13	25.2	CH_3	1.21, s	-	C6, C10, C11, C12
14	15.4	CH_3	1.30, d, 8.1	H7	C7, C8
15	23.7	CH_3	1.70, s	-	C2, C3, C4

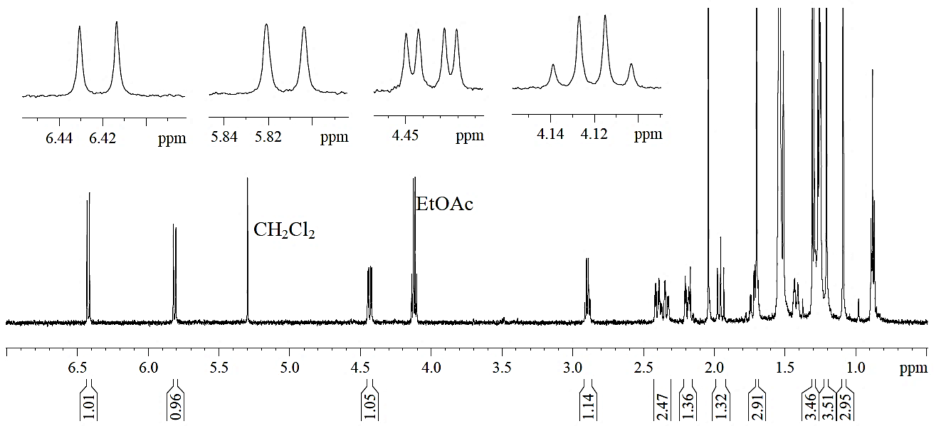

Figure S9. ^{1}H NMR spectrum ($CDCl_3$, 600 MHz) of compound **1a**.

1.3 Compound 1b

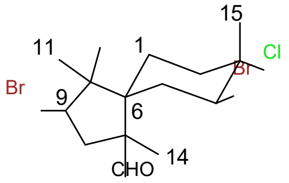

Table S2. NMR spectroscopic data of compound **1b**.

Carbon No	δ_C	δ_C mult	δ_H, mult, *J* (Hz)	COSY	HMBC
1	27.3	CH	1.70, m	H2a, H2b	-
2a	40.5	CH_2	2.32, td, 14.1, 4.0	H1	-
2b			2.42, dt, 14.1, 4.0	H1	-
3	71.0	C	-	-	-
4	60.5	CH	4.56, dd, 13.4, 3.6	H5a, H5b	-
5a	39.4	CH_2	1.92, t, 14.6	H4	-
5b			2.26, td, 14.6, 3.1	-	-
6	56.0	C	-	-	-
7	52.0	C	-	-	-
8a	43.4	CH_2	1.97, dd, 14.1, 8.4	H9	C7, C14
8b			2.88, dd, 14.1, 8.4	H8a, H9	C9, C13
9	58.4	CH	4.15, dd, 11.3, 8.4	H8a, H8b	C11
10	51.2	C	-	-	-
11	20.4	CH_3	0.88, s	-	C6, C9, C12
12	22.6	CH_3	0.98, s	-	C6, C9, C11
13	203.5	CHO	9.79, s	-	C6, C8, C14
14	23.2	CH_3	1.38, s	-	C6, C7, C8, C13
15	23.7	CH_3	1.71, s	-	C2, C3, C4

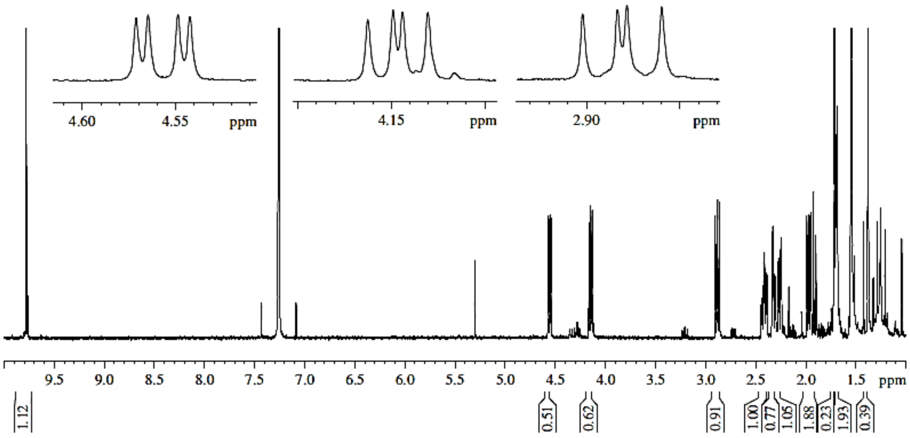

Figure S10. ^{1}H NMR spectrum ($CDCl_3$, 600 MHz) of compound **1b**.

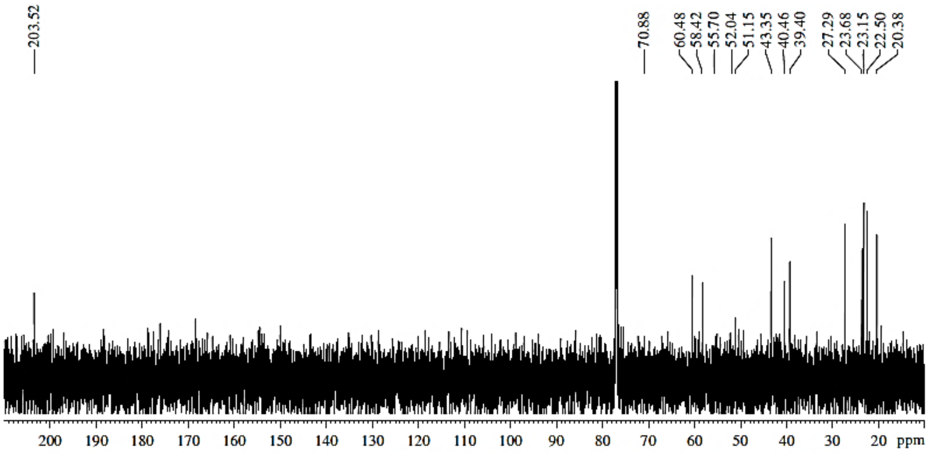

Figure S11. ^{13}C NMR spectrum ($CDCl_3$, 150 MHz) of compound **1b**.

1.4 Compound 2

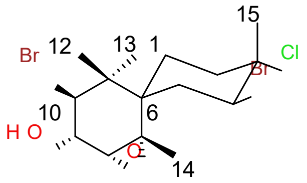

Table S3. NMR spectroscopic data of compound **2**.

Carbon No	δ_C	δ_C mult	δ_H, mult, J (Hz)
1	25.4	CH_2	2.06, dt, 14.6, 5.9
2a	40.0	CH_2	2.37, ddd, 14.6, 5.9, 2.1
2b			2.46, dd, 14.6, 5.9
3	70.5	C	-
4	61.9	CH	4.81, dd, 13.3, 5.4
5a	38.8	CH_2	2.18, m
5b			2.53, m
6	44.5	C	-
7	64.8	C	-
8	65.2	CH	3.04, s
9	70.7	CH	4.23, dd, 9.1, 2.8
10	69.9	CH	3.96, d, 9.1
11	42.9	C	-
12	28.5	CH_3	1.16, s
13	20.0	CH_3	1.16, s
14	25.5	CH_3	1.52, s
15	24.0	CH_3	1.74, s

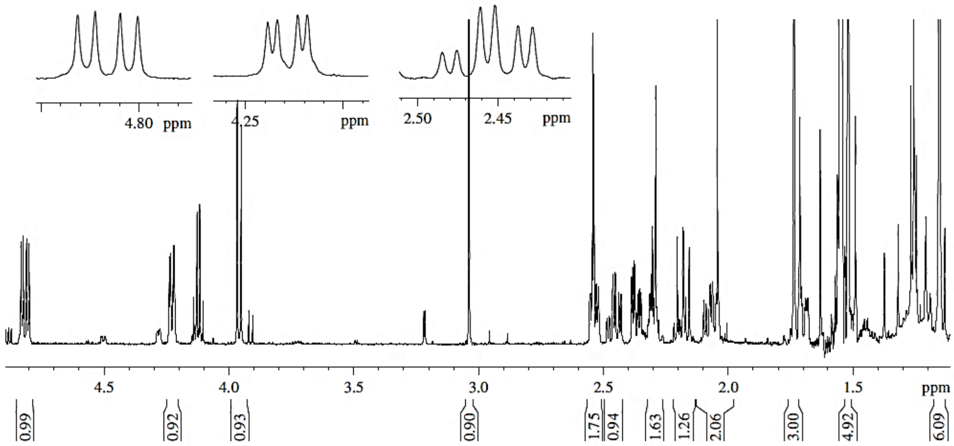

Figure S12. ^{1}H NMR spectrum ($CDCl_3$, 600 MHz) of compound **2**.

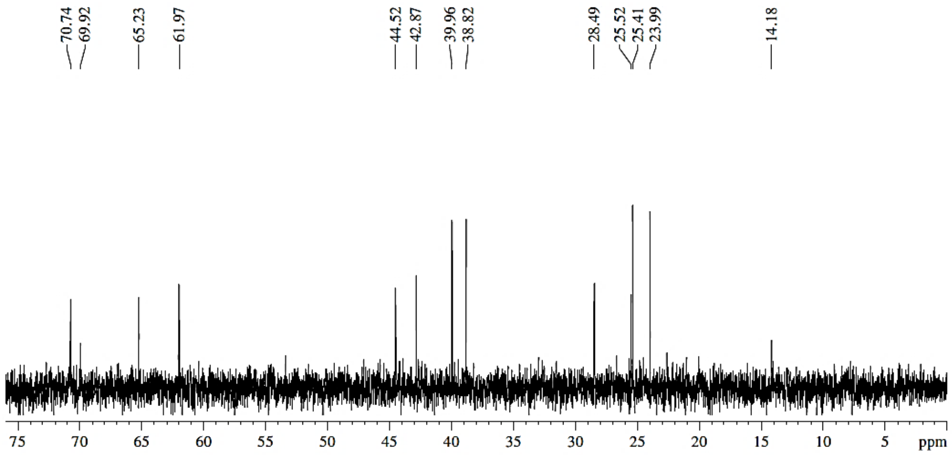

Figure S13. ^{13}C NMR spectrum ($CDCl_3$, 600 MHz) of compound **2**.

1.5 Compound 3

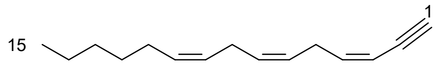

Table S4. NMR spectroscopic data of compound **3**.

**Carbon No**	◎ C[#]	◎ C **mult**	◎ H**, mult, *J* (Hz)**
1	81.8	CH	3.11, m
2	80.2	C	-
3	108.3	CH	5.48, m
4	143.7	CH	5.96, m
5	25.7	CH_2	2.84, m
6	125.8	CH	5.41, m
7	127.4	CH	5.36, m
8	27.3	CH_2	2.06, m
9	129.9	CH	5.45, m
10	130.6	CH	5.41, m
11	28.7	CH_2	3.11, m
12	29.3	CH_2	1.37, m
13	31.5	CH_2	1.28, m
14	22.5	CH_2	1.31, m
15	14.1	CH_3	0.89, t, 7.1

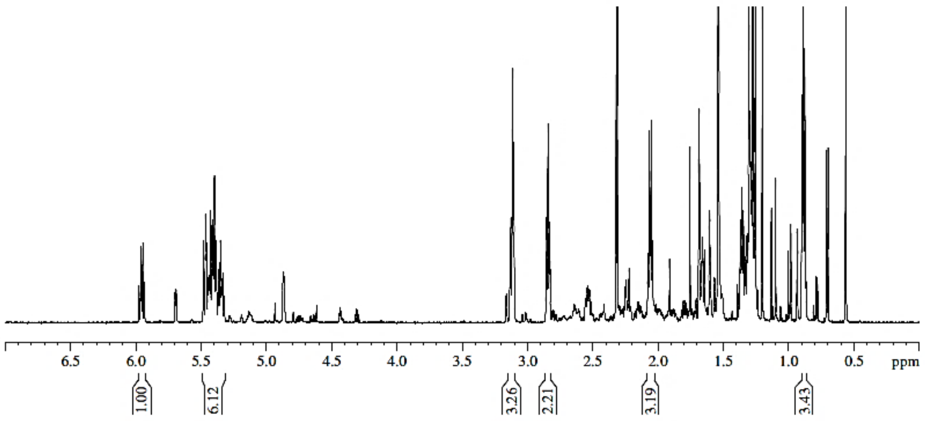

Figure S14. ^1H NMR spectrum ($CDCl_3$, 600 MHz) of compound **3**.

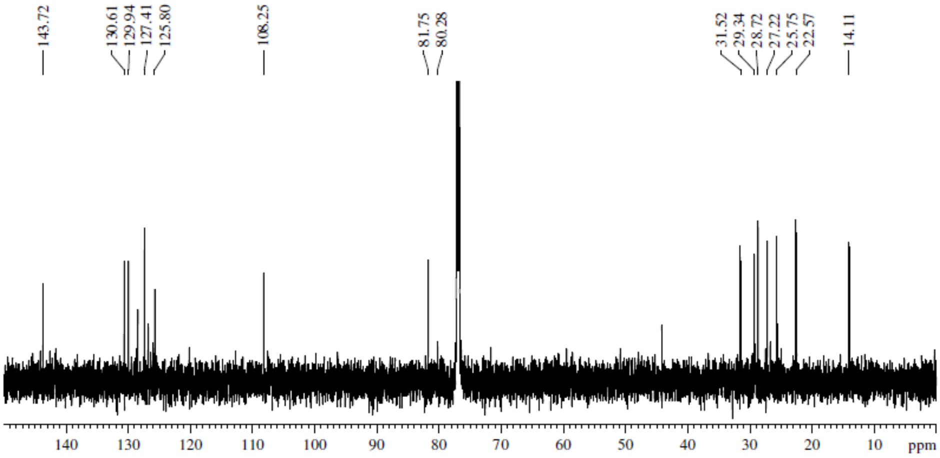

Figure S15. ^{13}C NMR spectrum ($CDCl_3$, 150 MHz) of compound **3**.

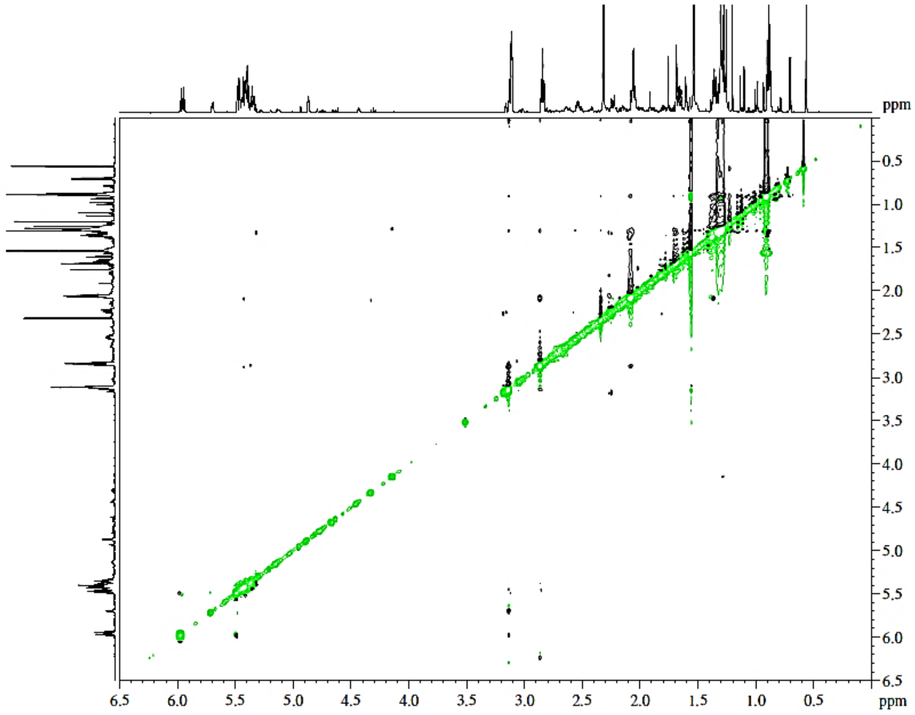

Figure S16. NOESY NMR spectrum ($CDCl_3$, 600 MHz) of compound **3**.

1.4 Compound 4

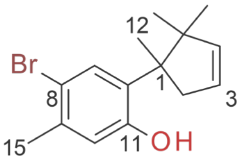

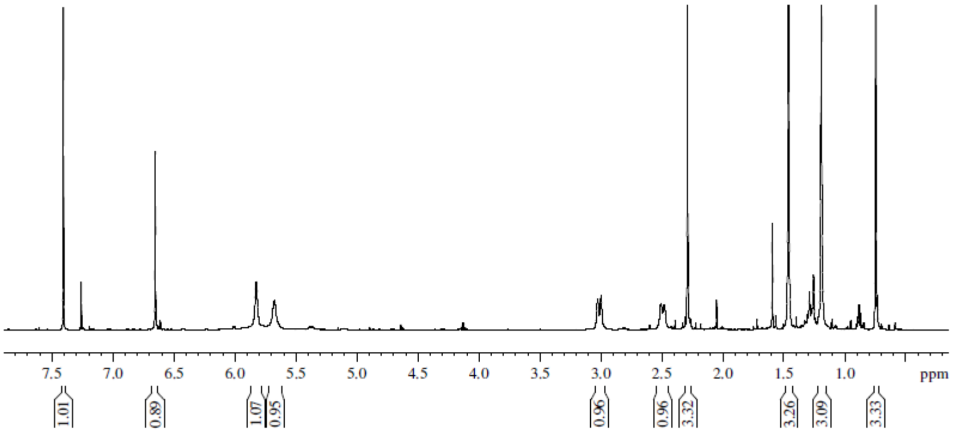

Figure S17. ^{1}H NMR spectrum ($CDCl_3$, 600 MHz) of compound **4**.

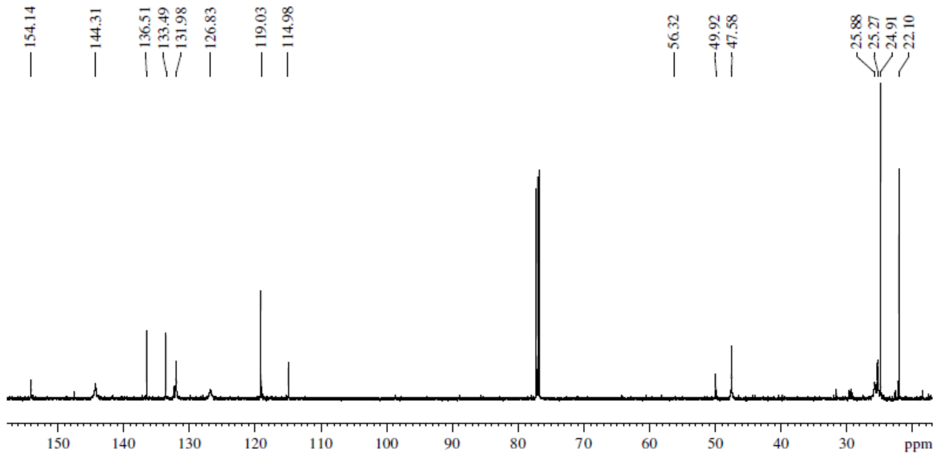

Figure S18. ^{13}C NMR spectrum ($CDCl_3$, 150 MHz) of compound **4**.

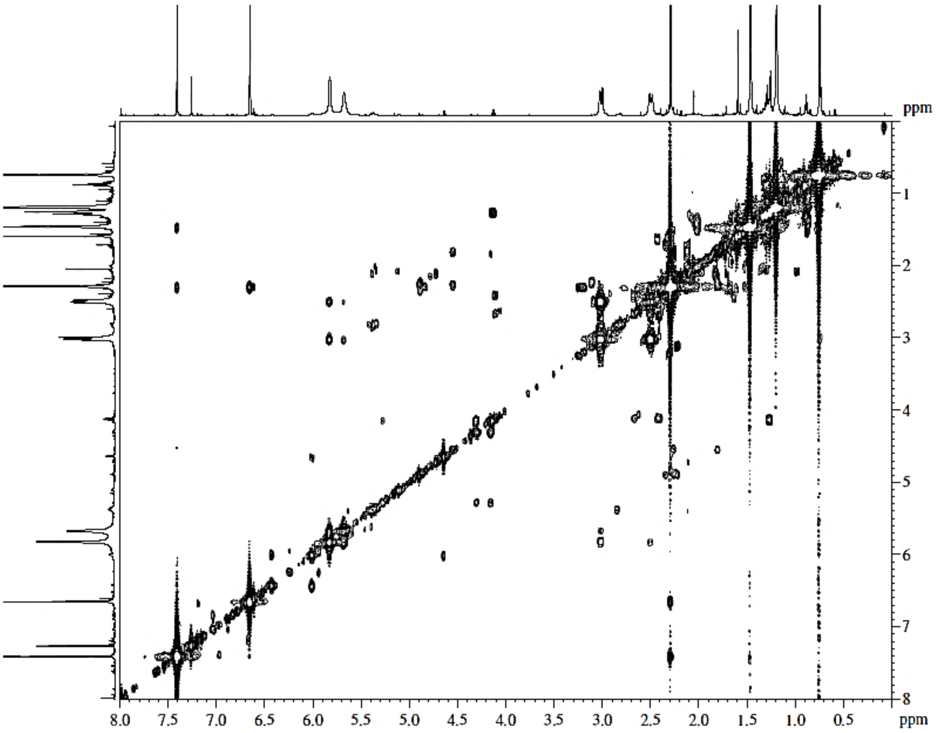

Figure S19. COSY NMR spectrum ($CDCl_3$, 600 MHz) of compound **4**.

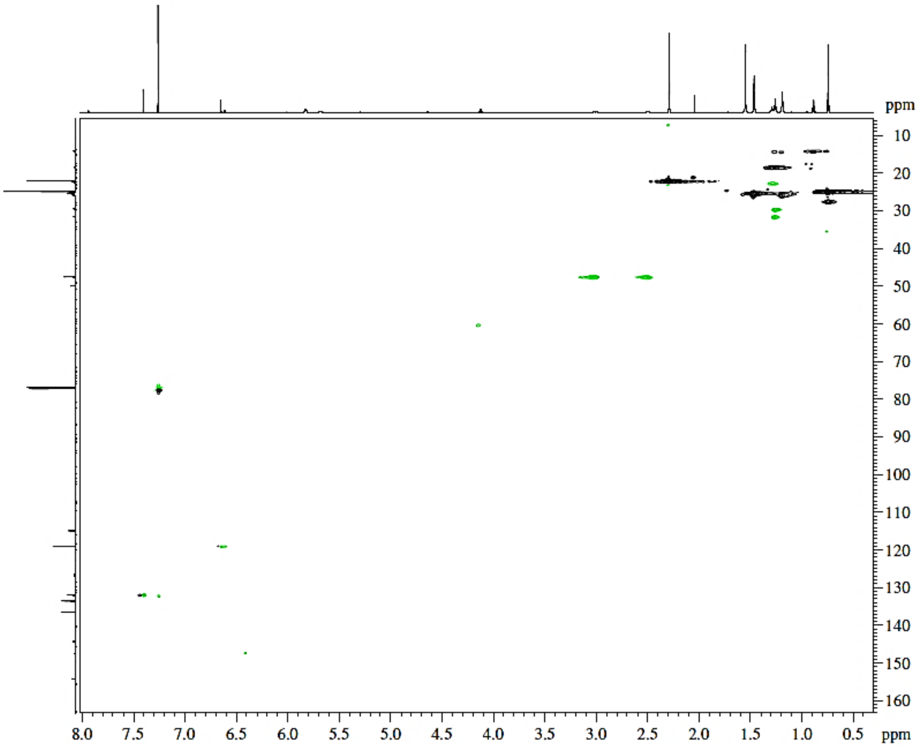

Figure S20. HSQC NMR spectrum ($CDCl_3$, 600 MHz) of compound **4**.

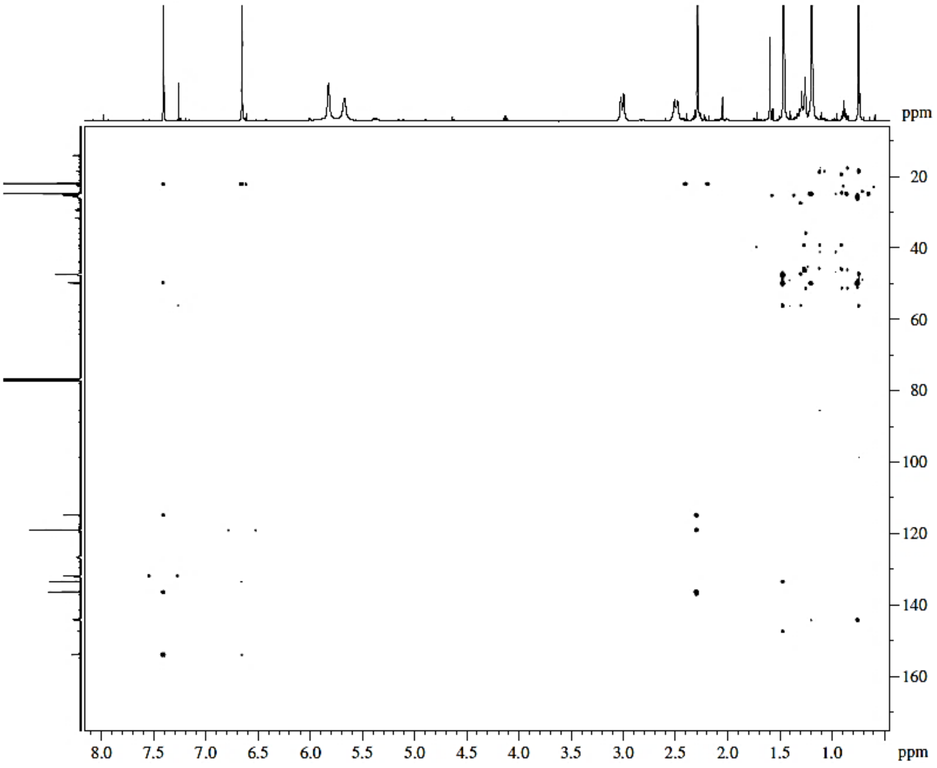

Figure S21. HMBC NMR spectrum ($CDCl_3$, 600 MHz) of compound **4**.

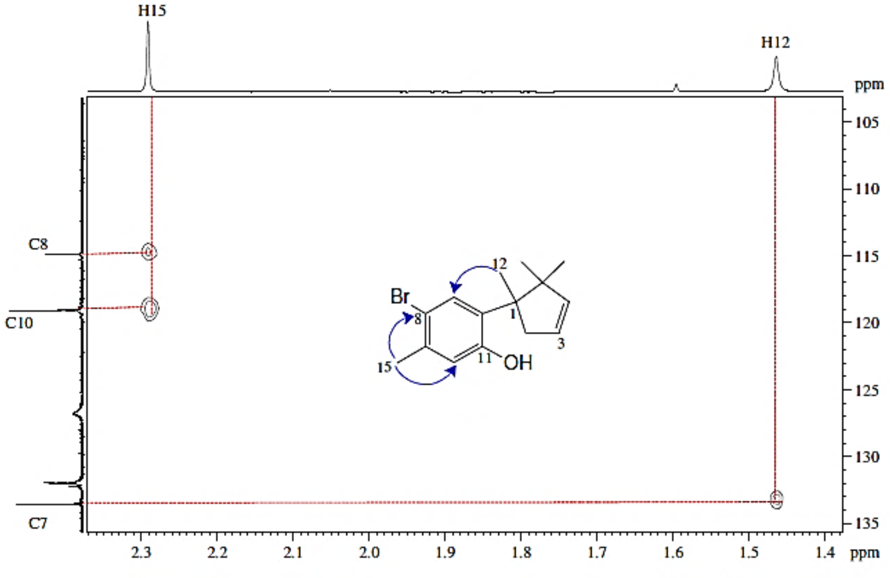

Figure S22. Partial HMBC spectrum of compound **4** showing key correlations.

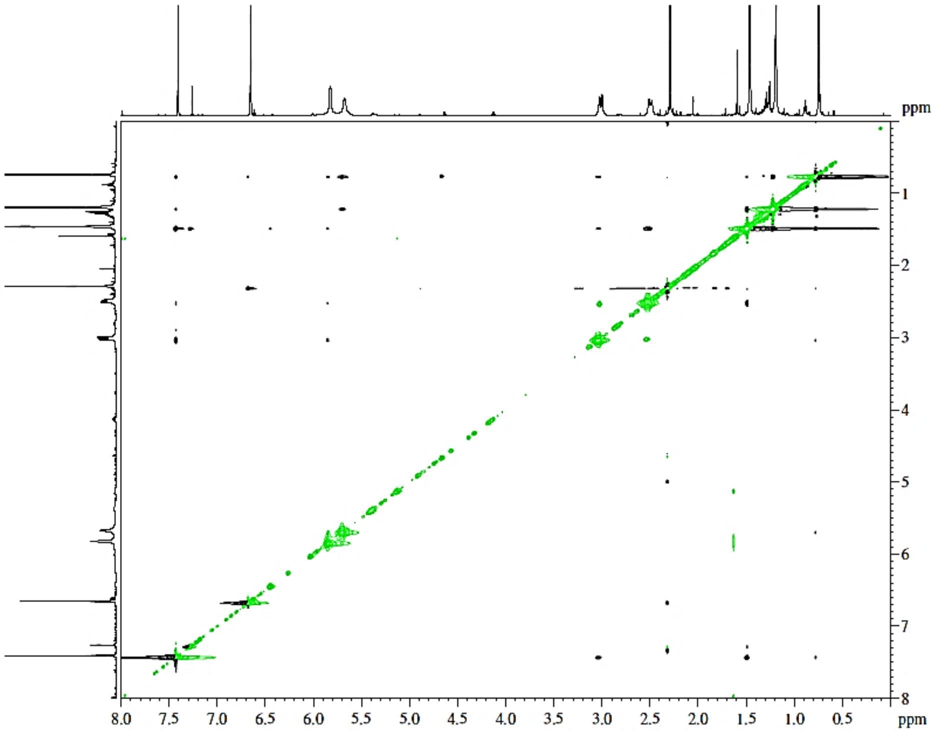

Figure S23. NOESY NMR spectrum ($CDCl_3$, 600 MHz) of compound **4**.

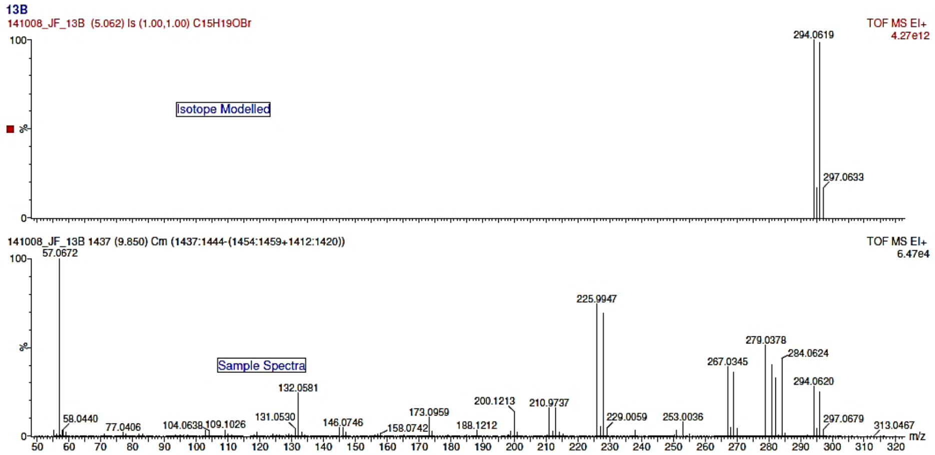

Figure S24. HRGCEIMS spectra of compound **4**.

1.5 Compound 4a

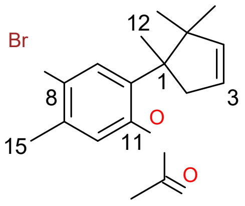

Table S5. NMR spectroscopic data of compound **4a**.

Carbon No	δC	δC mult	δH, mult, J (Hz)	COSY	HMBC
1	51.5	C	-	-	-
2a	45.7	CH_2	3.19, d, 16.1	H2b	C1, C3, C4, C6, C12
2b			2.30, m	H2a	-
3	125.3	CH	5.63, m	H2a, H2b, H4	C2, C4, C5
4	141.7	CH	5.39, dd, 5.9, 2.0	H2a, H2b, H3	C2, C3, C5
5	50.1	C	-	-	-
6	138.0	C	-	-	-
7	133.4	CH	7.63, s	-	C1, C8, C9, C11, C15
8	121.4	C	-	-	-
9	136.5	C	-	-	-
10	126.1	CH	6.84, s	H15	C6, C8, C11, C15
11	148.0	C	-	-	-
12	25.4	CH_3	1.28, s	-	C2, C5, C6
13	23.6	CH_3	1.29, s	-	C4, C5, C14
14	25.0	CH_3	0.75, s	-	C4, C5, C13
15	21.8	CH_3	2.33, s	H10	C8, C9, C10
16	169. 6	$OCOCH_3$	-	-	-
17	21.8	$OCOCH_3$	-	-	C16

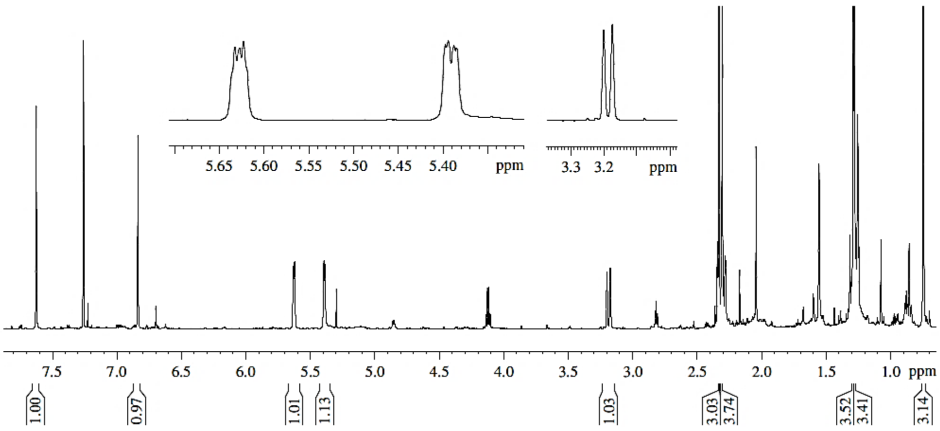

Figure S25. ^1H NMR spectrum ($CDCl_3$, 600 MHz) of compound **4a**.

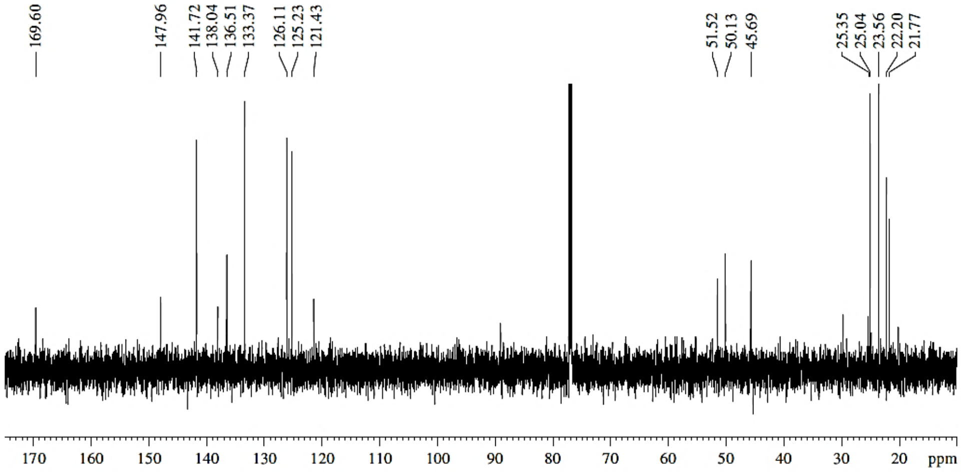

Figure S26. ^{13}C NMR spectrum ($CDCl_3$, 150 MHz) of compound **4a**.

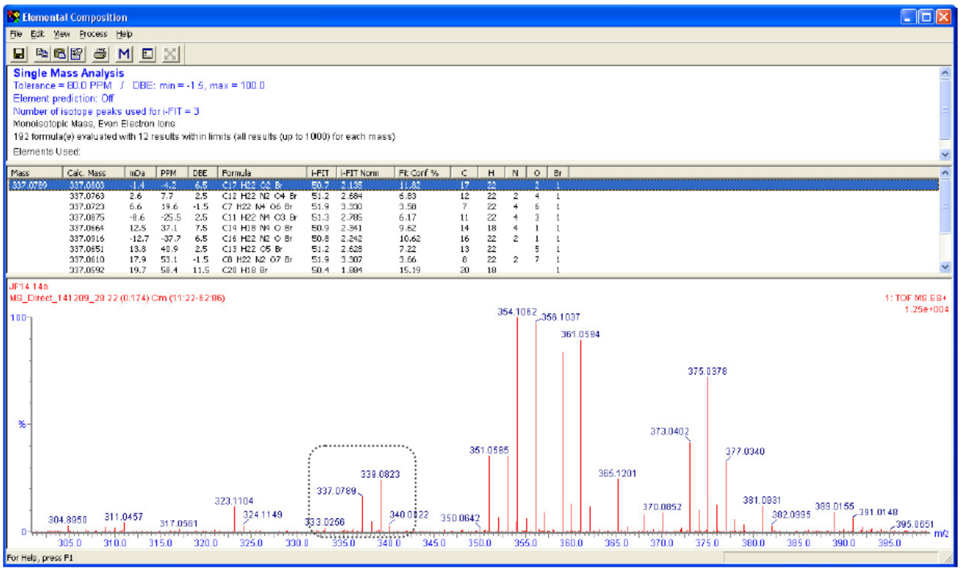

Figure S27. Expansion of the HRGCEIMS spectrum of compound **4a**.

1.6 Compound 5

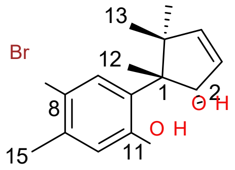

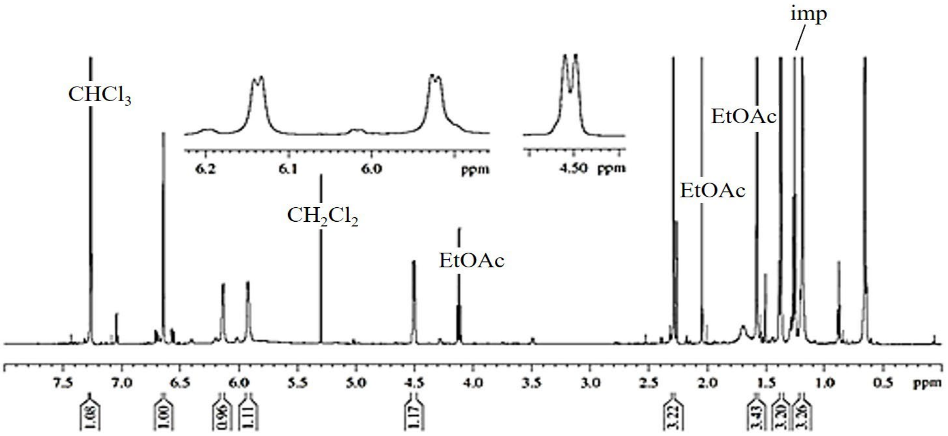

Figure S28. ^{1}H NMR spectrum ($CDCl_3$, 600 MHz) of compound **5**.

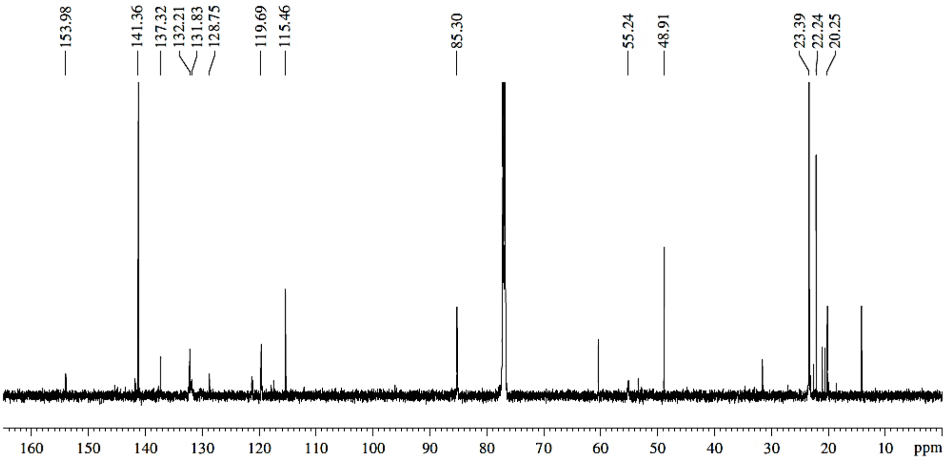

Figure S29. ^{13}C NMR spectrum ($CDCl_3$, 150 MHz) of compound **5**.

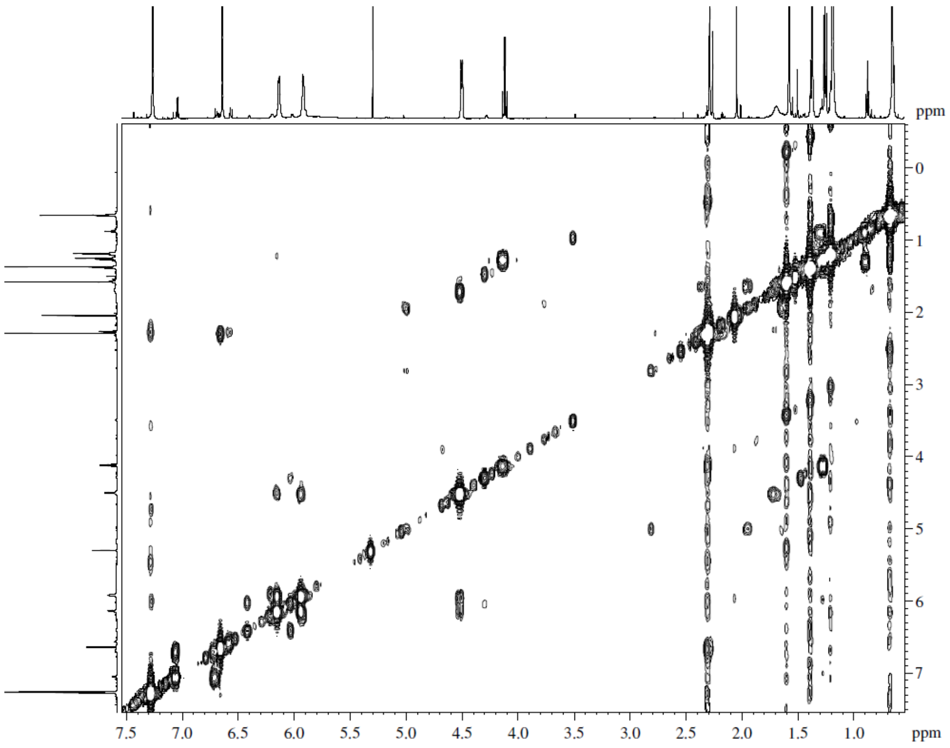

Figure S30. COSY NMR spectrum ($CDCl_3$, 600 MHz) of compound **5**.

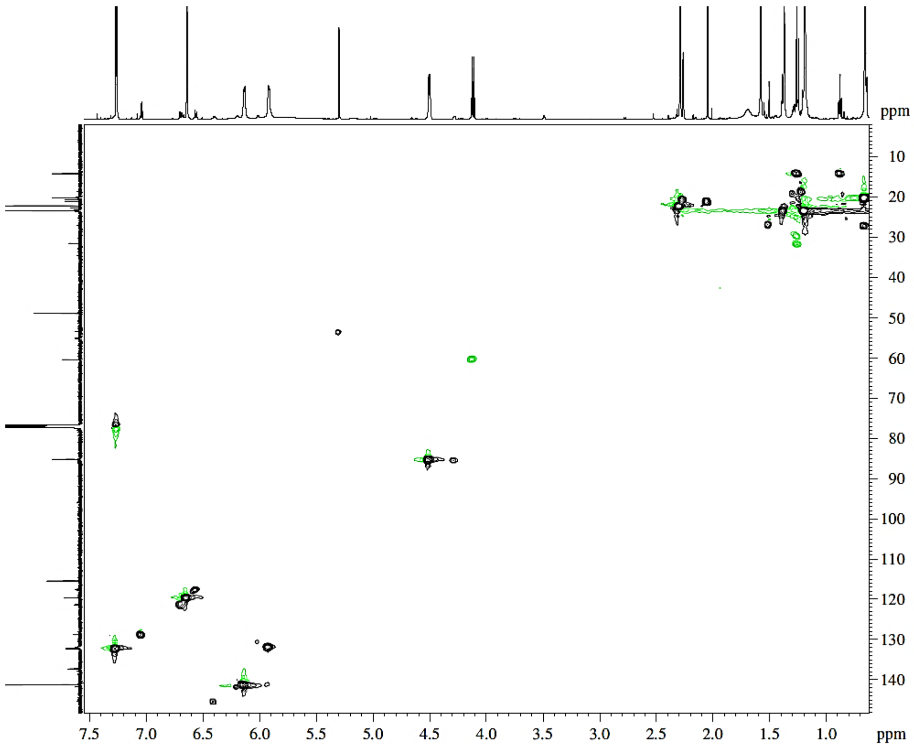

Figure S31. HSQC NMR spectrum ($CDCl_3$, 600 MHz) of compound **5**.

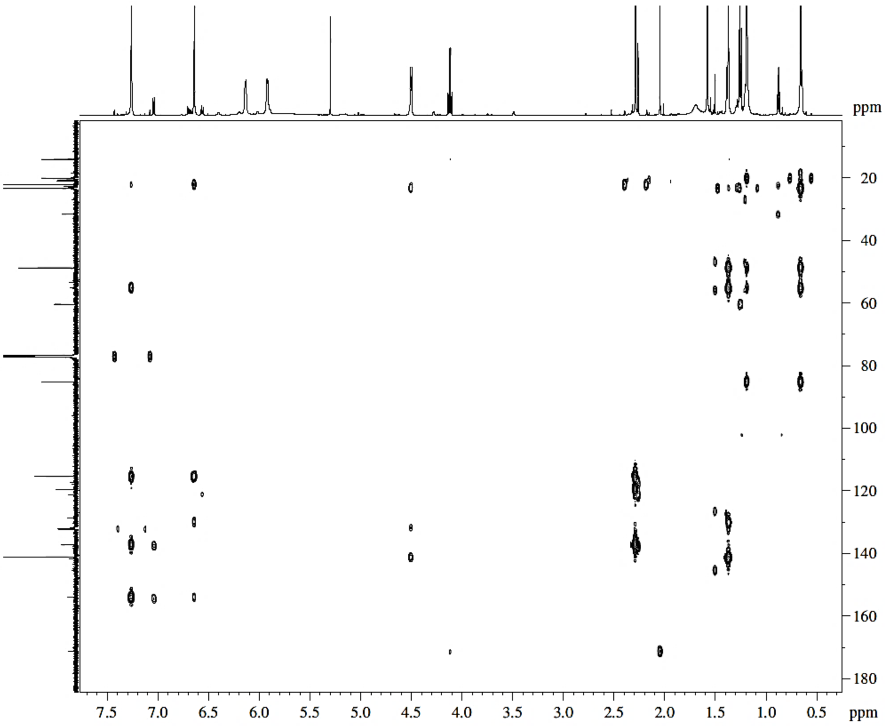

Figure S32. HMBC NMR spectrum ($CDCl_3$, 600 MHz) of compound **5**.

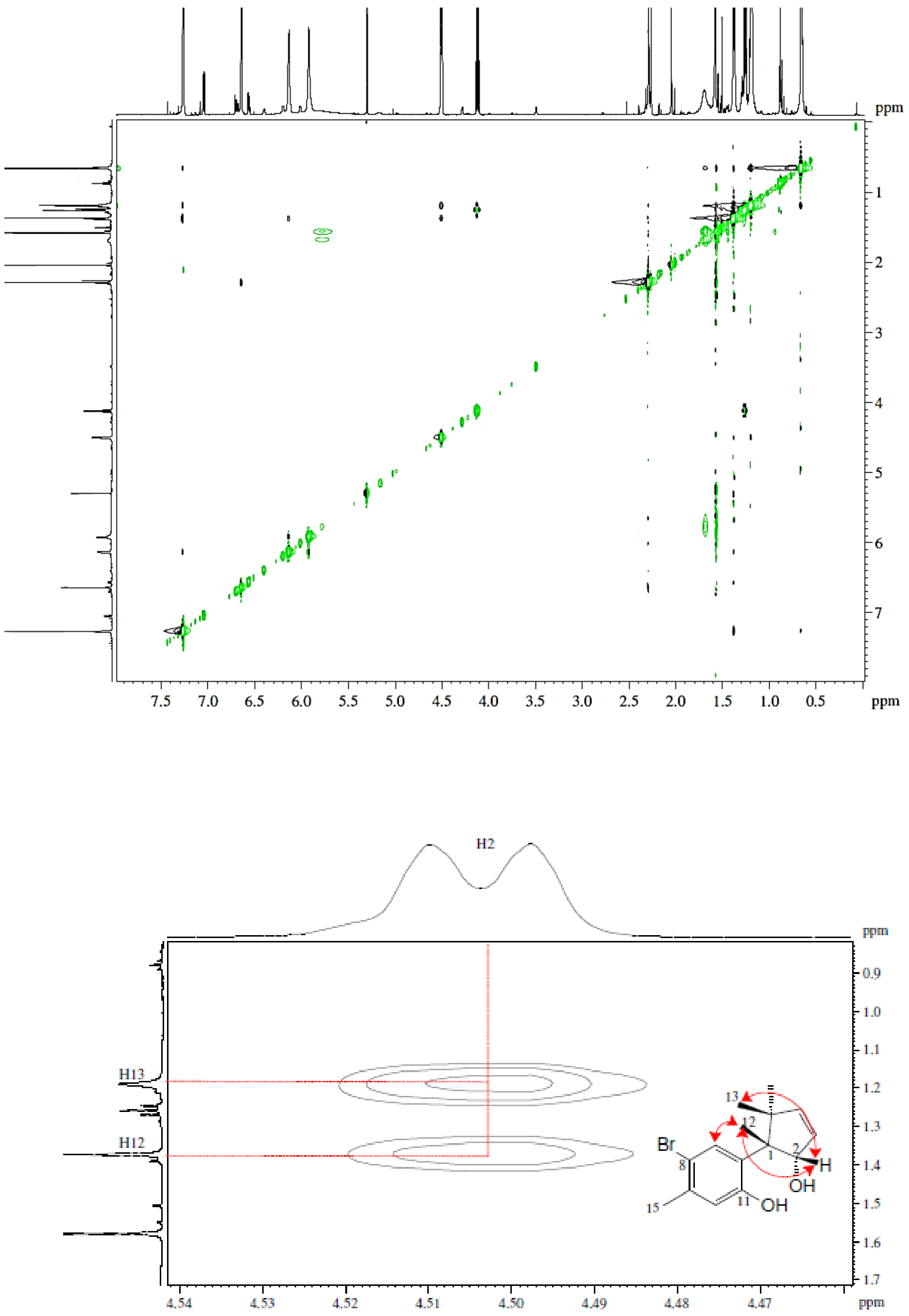

Figure S33. NOESY NMR spectrum ($CDCl_3$, 600 MHz) of compound **5**.

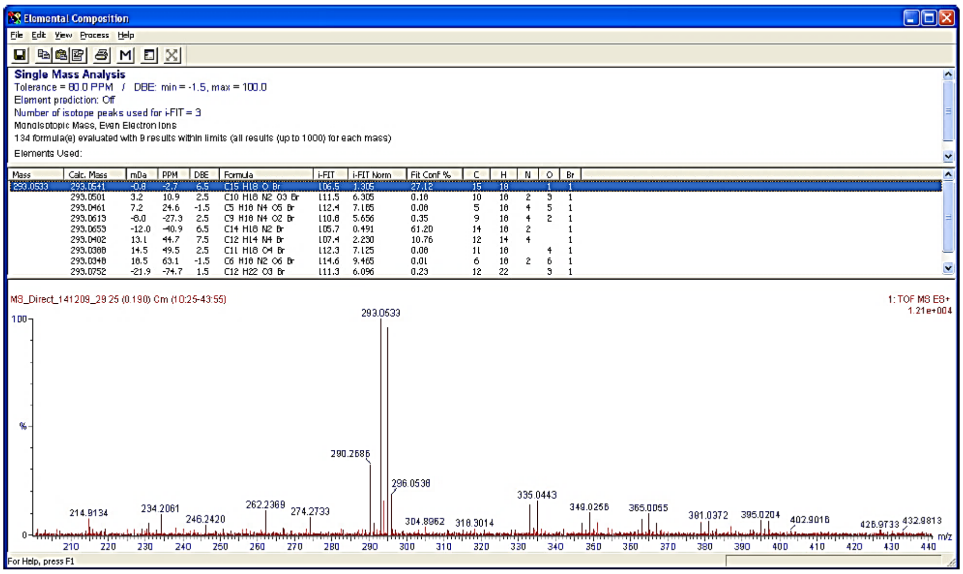

Figure S34. HRGCEIMS spectrum of compound **5.**

1.7 Compound 6

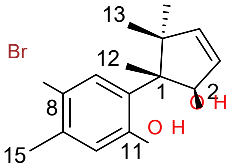

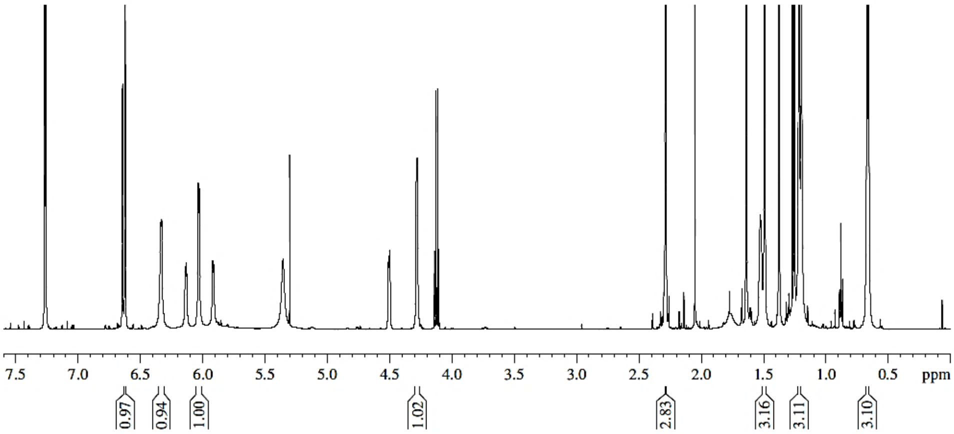

Figure S35. ^{1}H NMR spectrum ($CDCl_3$, 600 MHz) of compound **6**. Compound **6** was isolated as a mixture with compound **5**.

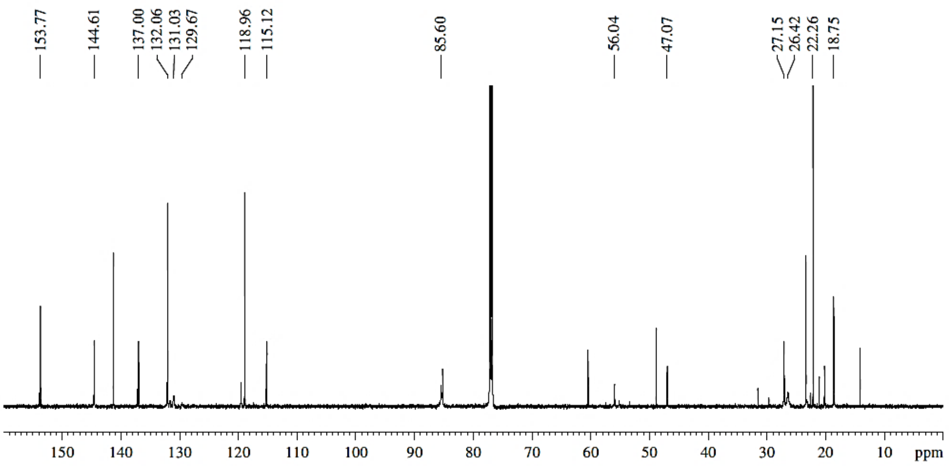

Figure S36. ^{13}C NMR spectrum ($CDCl_3$, 150 MHz) of compound **6**.

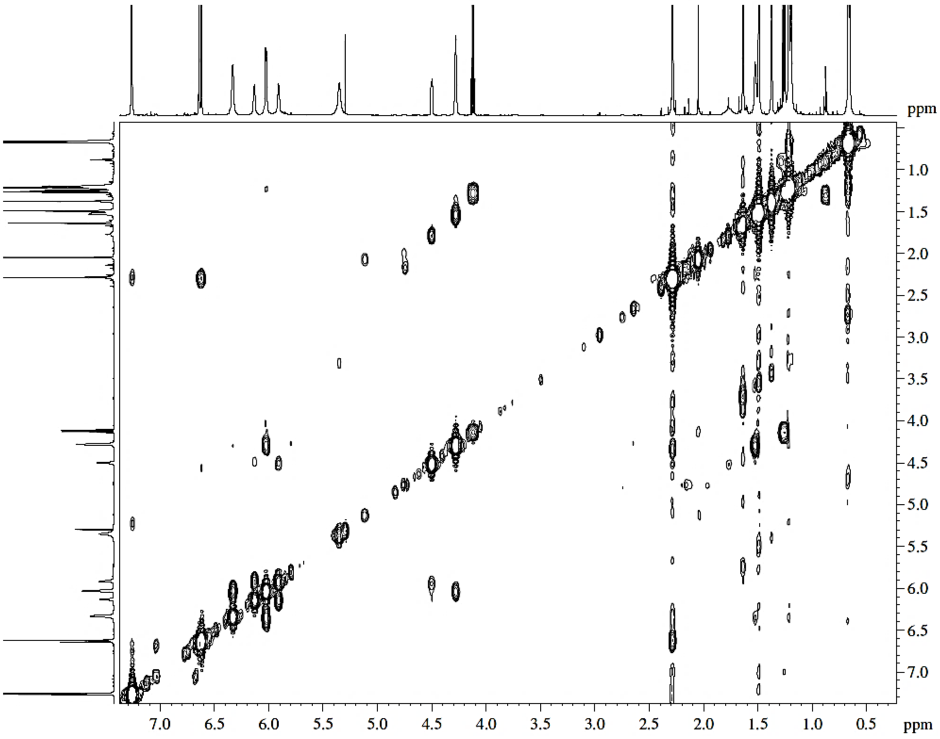

Figure S37. COSY NMR spectrum ($CDCl_3$, 600 MHz) of compound **6**.

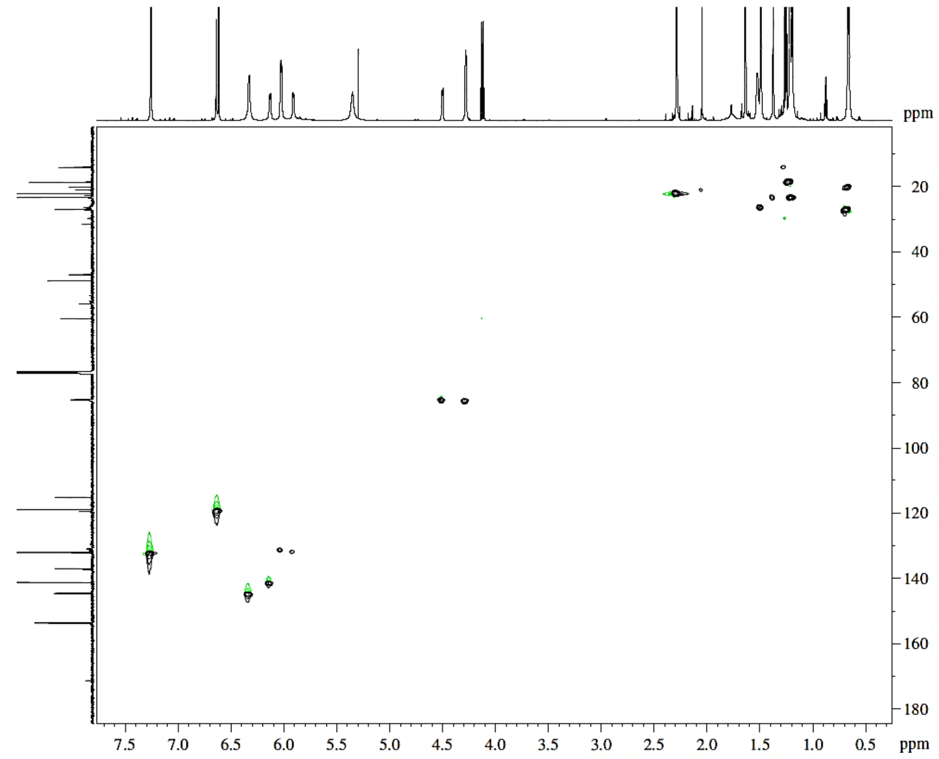

Figure S38. HSQC NMR spectrum ($CDCl_3$, 600 MHz) of compound **6**.

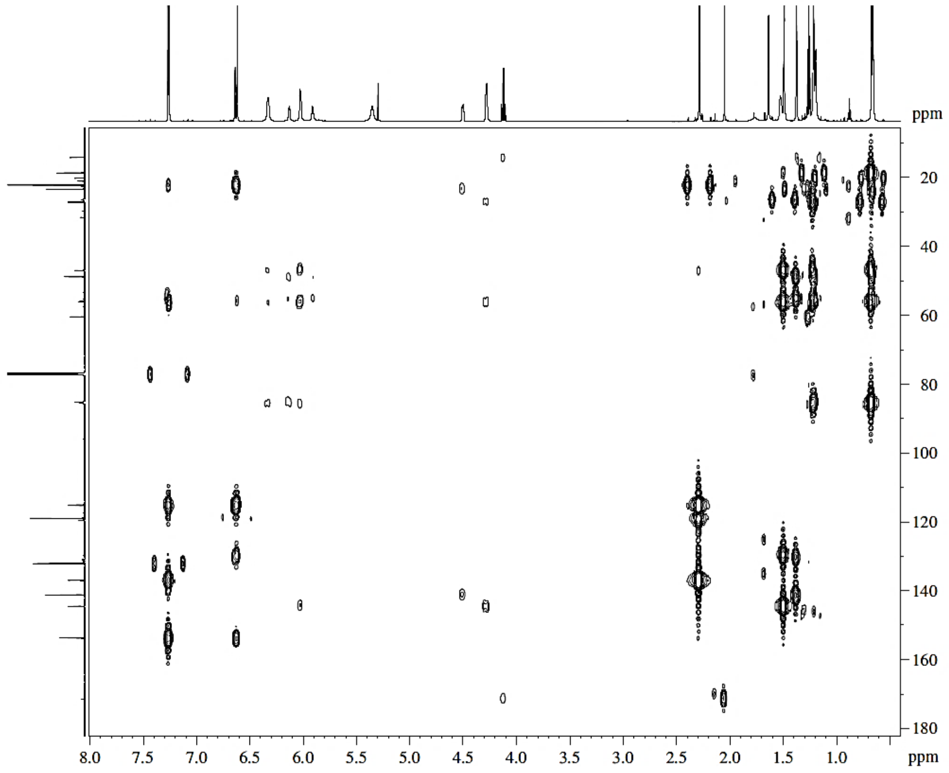

Figure S39. HMBC NMR spectrum ($CDCl_3$, 600 MHz) of compound **6**.

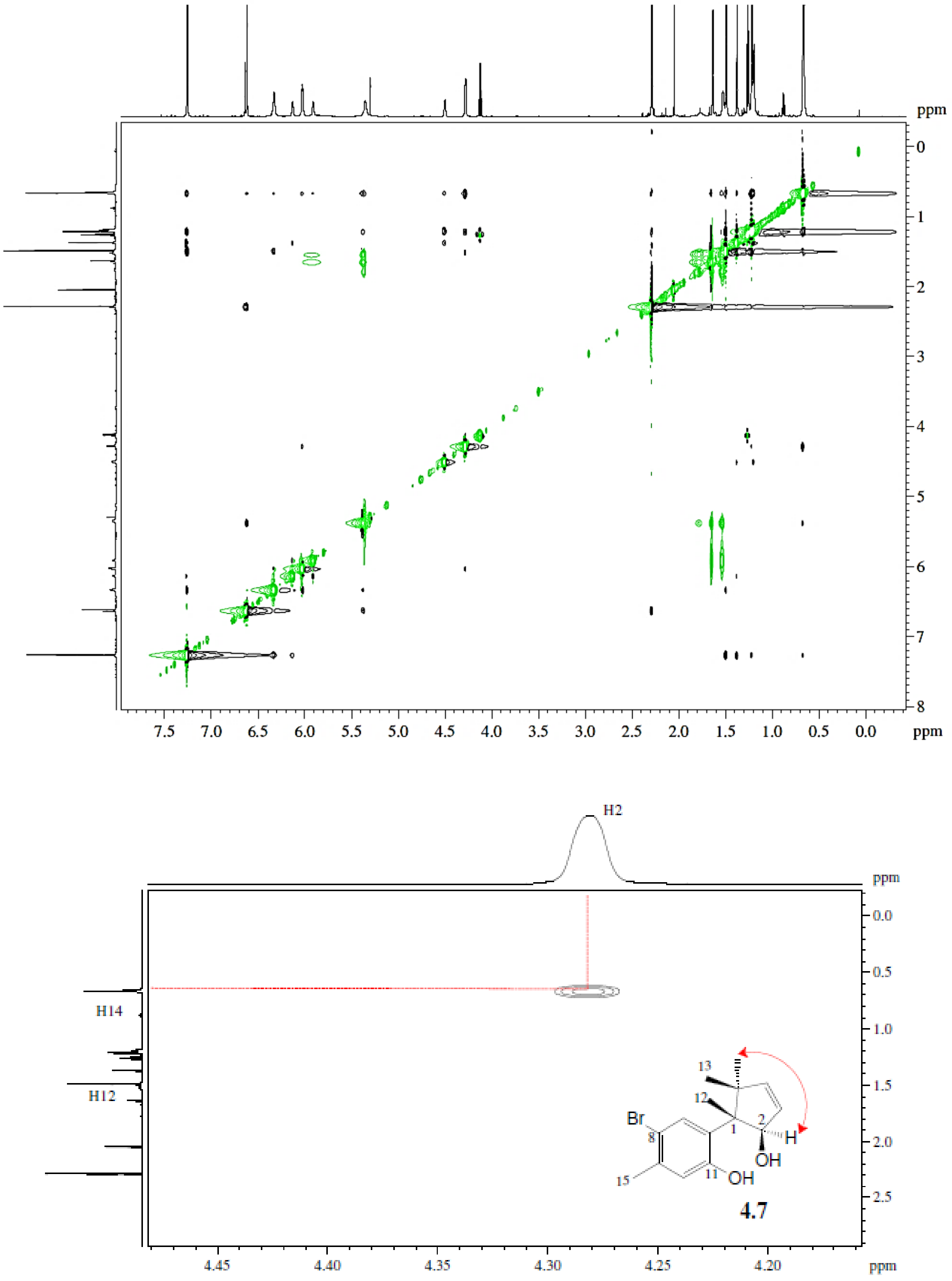

Figure S40. NOESY NMR spectrum ($CDCl_3$, 600 MHz) of compound **6**.

1.8 Compound 7

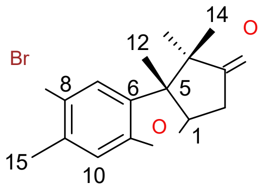

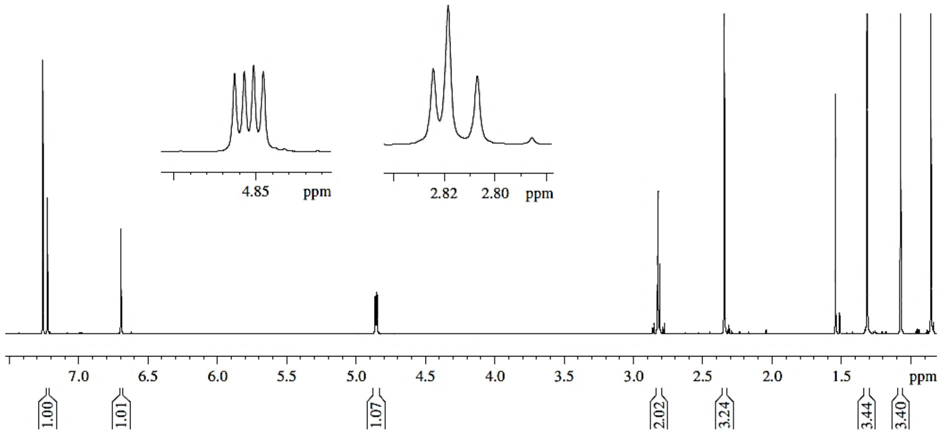

Figure S41. ^{1}H NMR spectrum ($CDCl_3$, 600 MHz) of compound **7**.

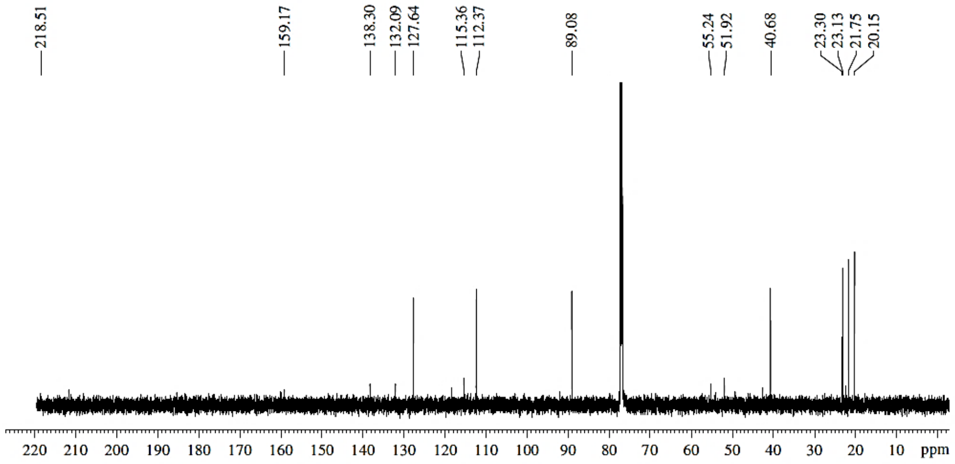

Figure S42. ^{13}C NMR spectrum ($CDCl_3$, 150 MHz) of compound **7**.

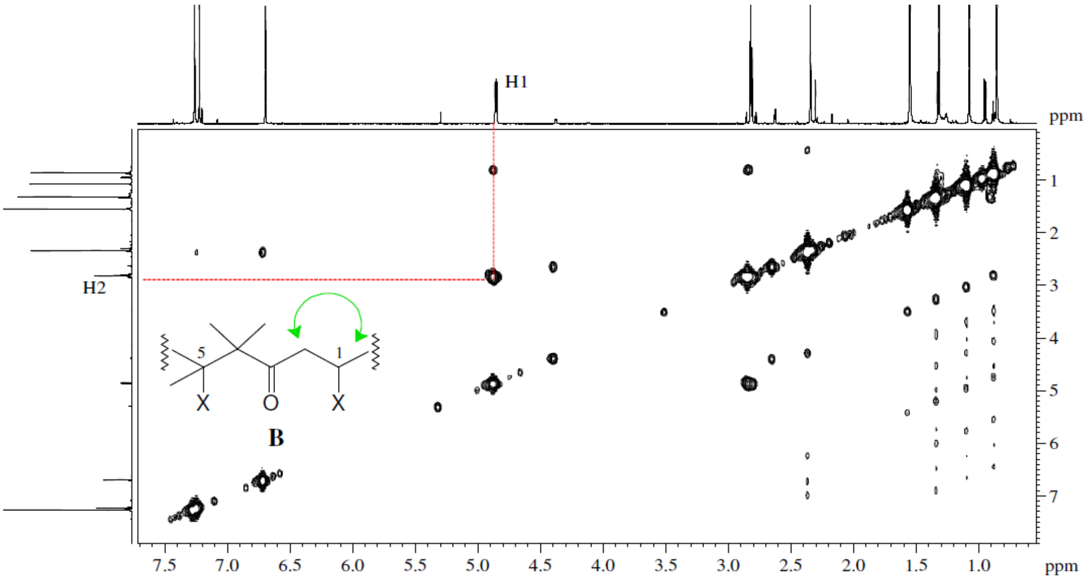

Figure S43. COSY NMR spectrum (CDCl3, 600 MHz) of compound **7**.

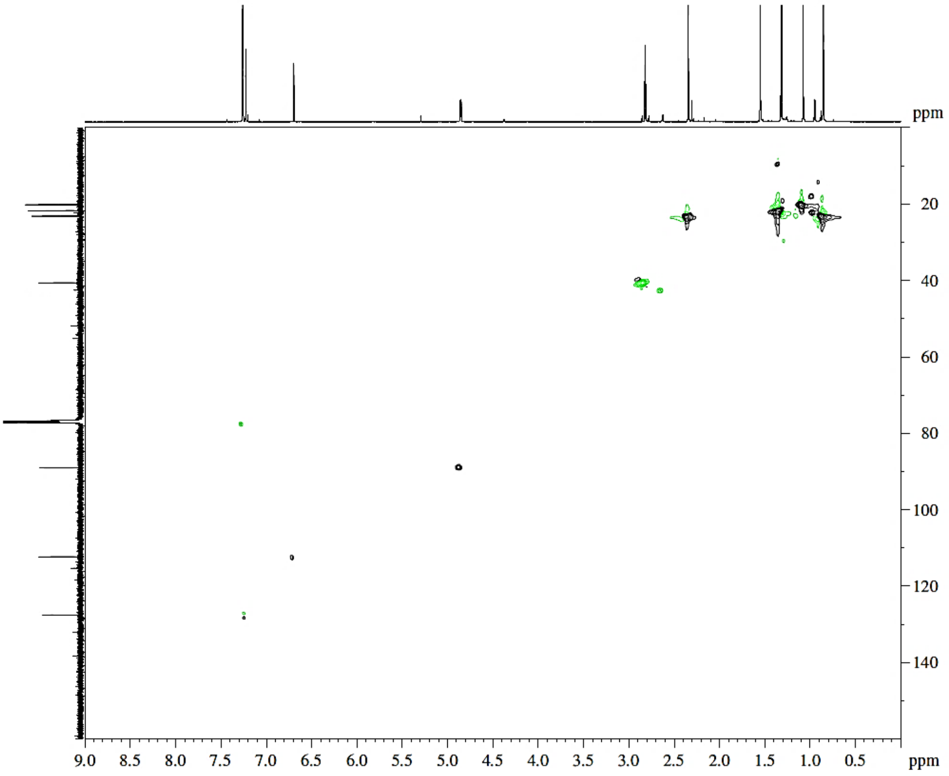

Figure S44. HSQC NMR spectrum (CDCl3, 600 MHz) of compound **7**.

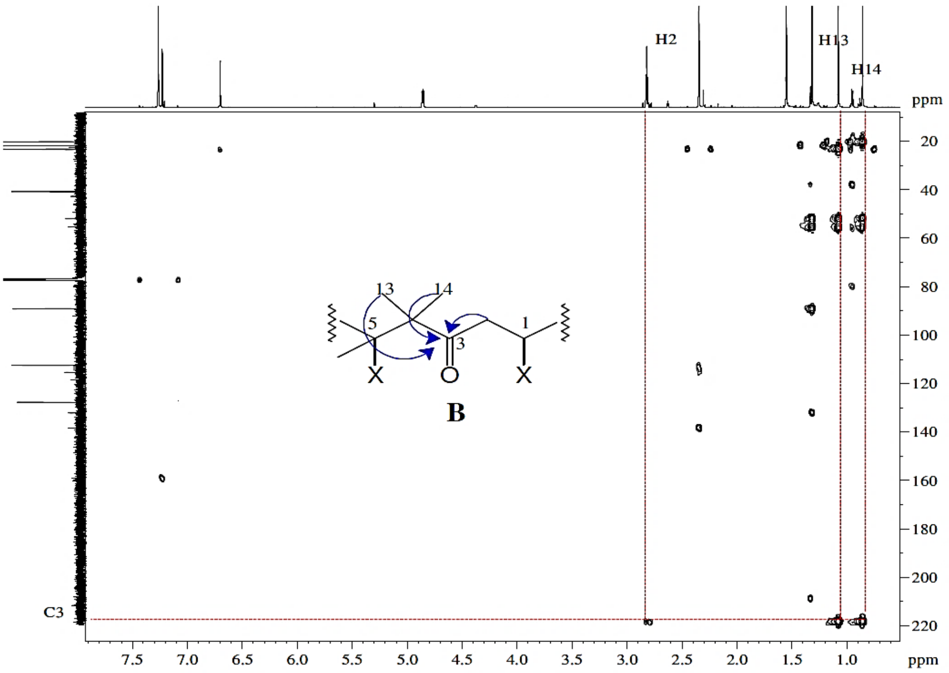

Figure S45. HMBC NMR spectrum ($CDCl_3$, 600 MHz) of compound **7**.

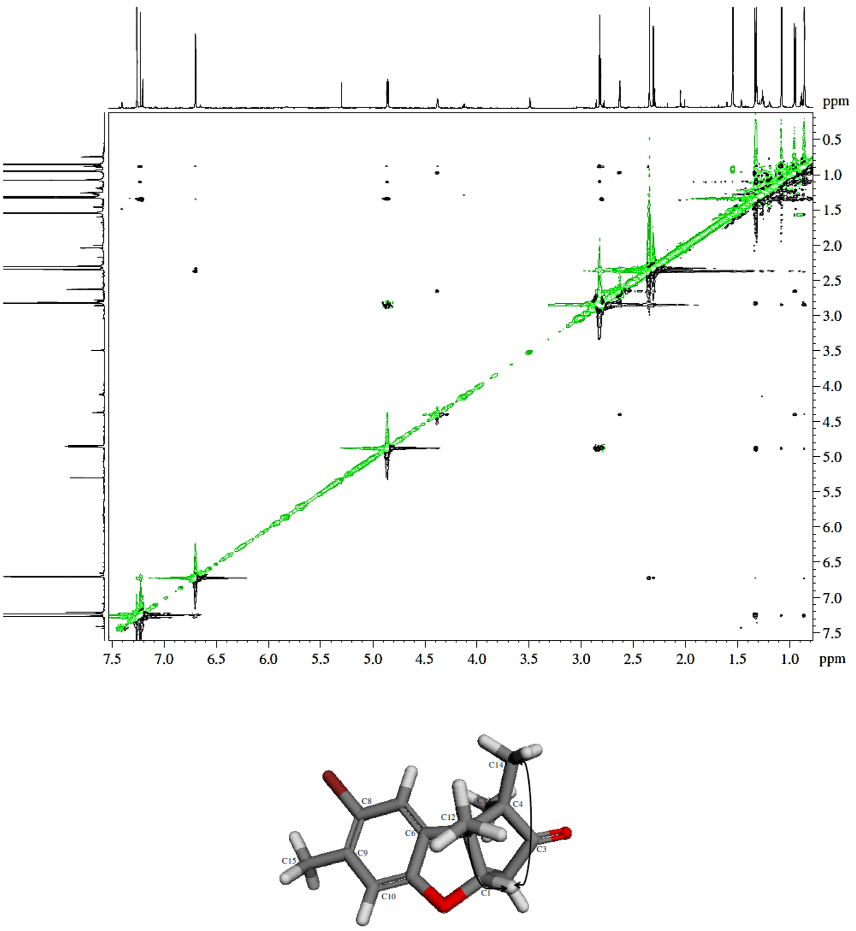

Figure S46. NOESY NMR spectrum ($CDCl_3$, 600 MHz) and key NOESY correlations for compound **7**.

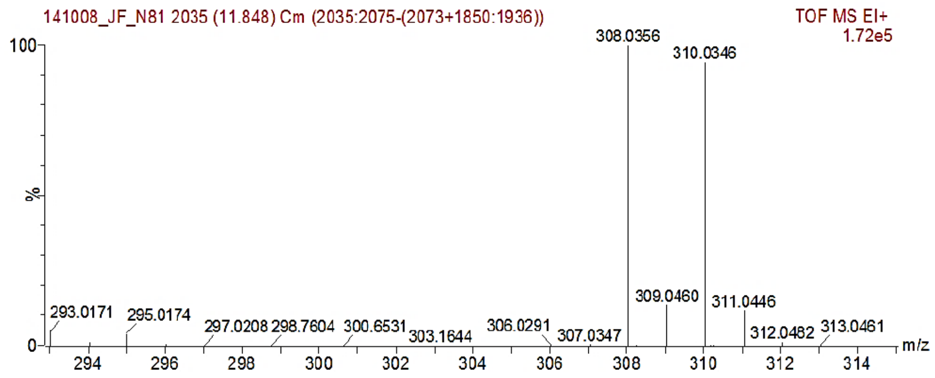

Figure S47. HRGCEIMS spectrum of compound **7**.

1.9 Compound 8

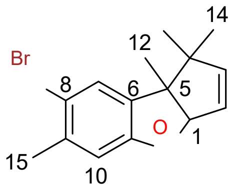

Table S6. NMR spectroscopic data of compound **8**.

Carbon No	◎ C#	◎ C mult	◎ H, mult, *J* (Hz)
1	99.3	CH	5.28, d, 1.8
2	124.8	CH	5.62, dd, 5.8, 1.8
3	147.6	CH	5.81, d, 5.8
4	52.0	C	-
5	54.7	C	-
6	133.8	C	-
7	129.0	CH	7.24, s
8	113.1	C	-
9	138.1	C	-
10	112.1	CH	6.66, s
11	157.8	C	-
12	22.3	CH_3	1.33, s
13	26.9	CH_3	1.07, s
14	27.1	CH_3	1.13, s
15	23.1	CH_3	2.31, s

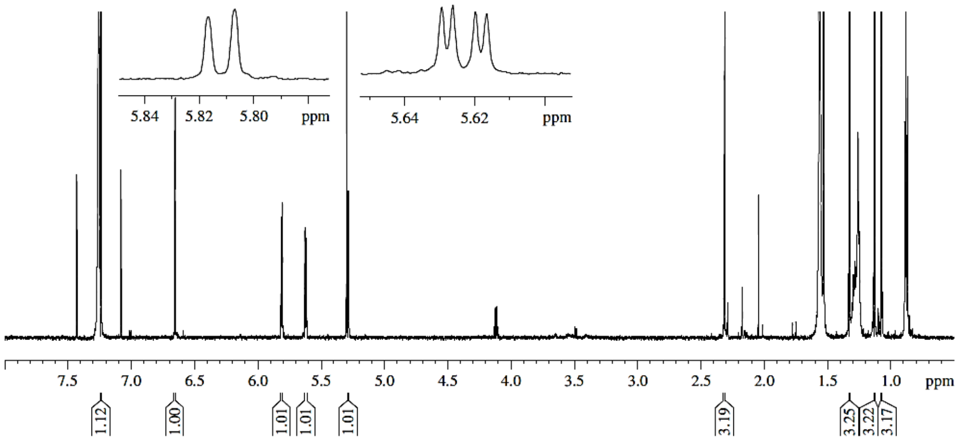

Figure S48. ^{1}H NMR spectrum ($CDCl_3$, 600 MHz) of compound **8**.
